# Process optimization of *Pediococcus pentosaceus* 4412-derived exopolysaccharides as a prebiotic in banana pseudostem juice fermentation

**DOI:** 10.3389/fnut.2026.1798892

**Published:** 2026-04-08

**Authors:** J. Angelin, V. Iswareya Lakshimi, Avinash Patnaik, Fathima Nowshad, P. Swetha, M. Kavitha

**Affiliations:** School of Bio Sciences and Technology, Vellore Institute of Technology, Vellore, Tamil Nadu, India

**Keywords:** banana pseudostem juice, exopolysaccharide, functional attributes, optimization, *P. pentosaceus* 4412, prebiotic, volatile compounds

## Abstract

**Introduction:**

Exopolysaccharide (EPS)-producing *Pediococcus pentosaceus* 4412 exhibits remarkable health-promoting properties, making it a promising candidate for functional food production.

**Methods:**

The production of EPS was statistically optimized using one-factor-at-a-time, Plackett-Burman, and response surface methodologies. CCD-based RSM optimized the prebiotic potential of EPS in the fermentation of banana pseudostem juice. The volatile organic compounds in fermented banana pseudostem juice (FBPJ) were analysed using GC-MS analysis. Further, the biological activity of FBPJ, including antimicrobial and antioxidant activity were assessed *in vitro*.

**Results and discussion:**

Primarily, EPS production was optimized using galactose (36.81 g/L), ammonium sulfate (2 g/L), and yeast extract (5 g/L), resulting in a 5.5-fold increase in yield (from the unoptimized baseline) to 3.46 ± 0.16 g/L. The prebiotic potential of EPS-4412 in the FBPJ was evaluated by monitoring viable probiotic cell counts, and the RSM optimized process resulted in a 3.67-fold increase in prebiotic activity. Viable cell counts were effectively maintained in the FBPJ17 sample compared with other samples after 30 days of storage at 4 °C. The pH and sugar contents decreased, whereas the total phenolic content (TPC) was elevated. The FBPJ17 exhibited significant antimicrobial activity against *Salmonella enterica* MTCC 1164 with a zone of inhibition of 15.7 ± 0.12 mm. The maximum DPPH and superoxide radical scavenging activities and reducing power of FBPJ17 were determined to be 74.43 ± 0.96%, 65.6 ± 1.1%, and 0.72 ± 0.058, respectively. In particular, EPS-4412 supported the growth of the lactic acid bacteria consortium (*Lactiplantibacillus pentosus* AKVIT2, *Lpb. plantarum* BFW MKVIT02, and *P. pentosaceus* 4412), which played a crucial role in the production of esters, ketones, and hydrocarbons, imparting fruity, floral, and citrus-like flavours. Hence, EPS-4412 could be considered as a prebiotic stimulant in industrial applications.

## 1 Introduction

Exopolysaccharides (EPSs), a postbiotic produced by probiotic bacteria during fermentation, exhibit prebiotic, antibacterial, antioxidant, and anti-inflammatory properties (1, 2). Prebiotics, notably EPS, promote the colonization of lactic acid bacteria (LAB) in the gut, protect beneficial microbes from the host immune response, augment their competitive advantage over harmful microorganisms, disrupt their adhesion to the intestinal epithelium, and inhibit or diminish pathogenic biofilm formation (3). EPS generated by *Lactobacillus reuteri* EC01 facilitated the multiplication of *Bifidobacterium longum* and *B. breve*, demonstrating prebiotic properties comparable to fructans (4). EPSs render substantial benefits, such as effortless production and storage, recovery in pure form, and unique biological functions (5). To address the challenge of low EPS yield, which impedes large-scale industrial exploitation, numerous strategies have been employed to maximize EPS production (6). EPS generation is strongly influenced by physicochemical parameters like pH, temperature, incubation period, inoculum size, and carbon and nitrogen sources (7). When several factors are evaluated, the traditional single-factor optimization technique becomes costly and time-consuming. Experimental statistical approaches, such as response surface methodology (RSM), are used to optimize fermentation conditions and overcome the disadvantages of single-factor optimization (8).

The use of bacterial fermentation to formulate functional foods with additional health benefits is generally acknowledged as an innovative approach to combating malnutrition. Several studies have recently focused on mixed LAB fermentation of fruits and vegetables, which is more successful than monoculture fermentation in enhancing their nutritional profile and reducing off-odor compounds that hamper consumer appeal (9). After 12 h of fermentation with *L. plantarum* LP56, the off-flavor of walnut milk was reduced and its functional qualities improved. This procedure promoted the quantities of sweet, umami, and vital amino acids, as well as unsaturated fatty acids, which contribute to a richer scent and nutritional profile (10). The beany flavor in unfermented mung beans, attributed to the presence of aldehydes such as nonanal, octanal, and butanal, was removed through effective fermentation using *L. plantarum* 23,169, which increased the production of acetic acid, ethyl hexanoate, and ethyl acetate, responsible for the fruity and grassy odor (11). The mixed LAB fermentation of jujube pulp with *L. plantarum*, *L. rhamnosus* GG, and *Streptococcus thermophilus* dramatically inhibited amino acid and lipid metabolism, whereas greater viable numbers and levels of aldehydes, alcohols, and ketones led to improved sensory properties (12).

The banana pseudostem exhibits significant health-promoting properties and contains phytochemicals, including tannins, alkaloids, phenolics, and flavonoids, which are more abundant than those in the fruit. It possesses remarkable therapeutic value but is rarely used in the human diet and often considered waste (13, 14). In a recent study, the banana pseudostem (BPS) core juice was fermented using *Saccharomyces ellipsoideus* NCIM-3200 and *L. plantarum* MTCC 6161 exhibited an extended shelf life (15). The most intriguing aspect of LAB-EPSs is their capacity to improve the texture, mouthfeel, and stability of foods, thereby allowing the development of innovative food products. Furthermore, EPS produced during fermentation plays an important role in enhancing the nutritional content and biological attributes of functional foods (16, 17). Fermentation in conjunction with EPS and LAB could encourage BPS juice intake and confer additional health benefits. The fermentation process employs RSM to identify optimal ranges for fermentation duration, substrate concentration, temperature, and inoculum size, rather than fixed values, hence facilitating personalized production of flavored foods (18).

The potential of probiotic-derived EPSs in fermentation for functional food development, particularly their synergistic usage by probiotic consortia, is largely untapped. The purpose of this study is to develop a symbiotic association between LAB, EPS, and banana pseudostem juice (BPS) through fermentation. The EPS used in the study was obtained from *P. pentosaceus* 4412, isolated from the fermented *Manilkara zapota* juice. The extraction, purification, and physicochemical characteristics of EPS-4412 were investigated. In our previous studies, promising anti-inflammatory activity of EPS-4412 was observed from *in vitro* analysis in RAW264.7 macrophages (LPS-induced), which was attributed to its structural characteristics (19). The present study aimed to optimize EPS production by *P. pentosaceus* 4412 through one-factor-at-a-time, Plackett-Burman, and response surface methodology. Further, the functional applications of EPS in enhancing probiotic fermentation of banana pseudostem juice through statistical optimization were investigated. The fermented product was evaluated for its physicochemical characteristics and bioactive properties.

## 2 Materials and methods

### 2.1 Chemicals and materials

2,2-diphenyl-1-picrylhydrazyl (DPPH), Potassium ferrocyanide, MRS agar, Mueller-Hinton broth, ascorbic acid, crystal violet dye, and ferric chloride were procured from HiMedia, India. DMSO, Trichloroacetic acid, acetic acid, and methanol were purchased from SRL.

### 2.2 Strains

Three strains, such as *P. pentosaceus* 4412, *L. plantarum* strain BFW MKVIT02, and *L. pentosus* AKVIT2, were isolated from fermented *Manilkara zapota*, *Musa acuminata* flower, and multigrain batter, respectively. The strains were identified by 16S rRNA gene sequencing, and the corresponding sequences were deposited in the NCBI GenBank repository under accession numbers OR095723, OR088203, and PV603401. All strains were grown in MRS broth for each experiment, and the consortium was prepared by mixing equal volumes of the three strains. EPS-4412 extracted from *P. pentosaceus* 4412 was employed as a substrate to enhance both the fermentation process and the growth of the selected three LAB strains (19). The pathogens, including *Shigella flexneri* MTCC 1457, *Salmonella enterica* MTCC 1164, and *Bacillus cereus* MTCC 1272, were obtained from the Microbial Type Culture Collection (MTCC) repository in Chandigarh, India.

### 2.3 Enhanced EPS production through optimization

EPS production was optimized through a three-step approach, including univariate analysis and statistical designs, namely Plackett-Burman (PB) and response surface methodology (RSM). Univariate analysis was conducted as the first step to identify the optimum conditions and medium components for the production of EPS-4412. It was followed by PB analysis to identify the most significant factors influencing EPS production, and further, the interaction effects between significant factors and their optimum levels were analyzed through RSM.

#### 2.3.1 Univariate / OFAT analysis

The growth media and culture conditions were initially optimized using one-factor-at-a-time (OFAT) analysis. In the beginning, pH values (5.5, 6.0, 6.5, 7.0, 7.5, 8.0, and 8.5), temperature (25 °C, 35 °C, and 45 °C), incubation time (24, 48, 72, 96, and 120 h), and inoculation size (2, 4, 6, 8, and 10%) were calibrated. The medium’s components, encompassing sources of carbon (glucose, mannose, fructose, rhamnose, as well as galactose) and sources of nitrogen (peptone, tryptone, soya peptone, yeast extract, and casein), were optimized by incorporating the optimum pH, inoculum size, temperature, and fermentation time (20). After the incubation period, the culture broth was centrifuged at 8000 rpm for 15 min, 4 °C, and twice the volume of ice-cold absolute ethanol was added to the recovered supernatant to precipitate EPS. Then, the precipitated EPS was dissolved in sterile MilliQ water to estimate the overall carbohydrate content in EPS using the phenol-sulfuric acid technique with D-glucose as the reference (19, 21).

#### 2.3.2 Plackett-Burman statistical analysis

The PB experimental design is a cost-effective and efficient two-level experimental method used to identify significant parameters for enhancing EPS production (22). Based on the univariate analysis results, nine medium components (including two dummy factors) were considered for the PB analysis, involving 12 experimental runs. All variables at two levels (high and low) were represented as +1 and −1. The EPS (g/L) concentration was calculated as the response value. PB matrix and concentration of EPS (g/L) are presented in Table 1.

**Table 1:** The Plackett-Burman design matrix with EPS concentration.

Run order	A: Galactose	B: Peptone	C: Yeast extract	D: Tween 80	E: (NH₄)₂SO₄	F: CH₃COONa	G: MgSO₄	H: MnSO₄	J: K_2_HPO_4_	EPS concentration (g/L)
1	2	10	0.5	1	2	5	1	0.05	2	0.95 ± 0.26
2	20	10	5	0.1	0.2	0.5	1	0.02	2	1.86 ± 0.16
3	2	10	5	1	0.2	0.5	0.05	0.05	0.02	1.27 ± 0.18
4	2	1	5	0.1	2	5	0.05	0.05	2	0.99 ± 0.27
5	2	1	0.5	1	0.2	5	1	0.02	2	0.64 ± 0.11
6	20	10	0.5	1	2	5	0.05	0.02	0.02	1.83 ± 0.28
7	20	1	5	1	0.2	5	1	0.05	0.02	1.75 ± 0.25
8	20	1	5	1	2	0.5	0.05	0.02	2	1.93 ± 0.26
9	20	10	0.5	0.1	0.2	5	0.05	0.05	2	1.33 ± 0.14
10	20	1	0.5	0.1	2	0.5	1	0.05	0.02	2.11 ± 0.27
11	2	1	0.5	0.1	0.2	0.5	0.05	0.02	0.02	0.83 ± 0.09
12	2	10	5	0.1	2	5	1	0.02	0.02	1.34 ± 0.19
13	OFAT optimized medium									1.23 ± 0.11

The following first-order polynomial model was used in the PB experimental design and calculated by Equation 1.


$$Y=\beta_{0}+\sum_{i=1}^{k}\beta_{i}X_{i}$$ (1)

In which *Y* denotes the EPS concentration, *β_0_* represents the model intercept, *k* denotes the number of factors, *β_i_* indicates the linear coefficient, and *X_i_* expresses the independent variable. The PB design was developed by Design Expert software (Version 13.0, Stat-Ease Inc., Minneapolis, MN, United States), followed by the calculation of first-order polynomial coefficients. At a 95% confidence level (*p* < 0.05), three significant variables were selected for further statistical analysis (23).

#### 2.3.3 Response surface methodology

RSM provides a standard strategy for optimizing various parameters along with identifying interactions among different factors to increase the production of EPS (24). Based on PB analysis, three significant factors, namely galactose, ammonium sulfate, and yeast extract, were chosen for a five-level, three-factor central composite design (CCD) to strengthen EPS production. The CCD experimental design included 20 runs and six repeating center points for calculating a pure error of the sum of squares. The components were categorized into five levels, including low, medium, high, and axial points (−1, 0, +1, −*α*, and +α). A 20-run CCD design matrix for EPS production is represented in Table 2.

**Table 2:** CCD design matrix for EPS production by *P. pentosaceus* 4412.

Run order	A: Galactose (g/L)	B: Ammonium sulfate (g/L)	C: Yeast extract (g/L)	EPS concentration (g/L)	
				Experimental value	Predicted value
1	20	2	5	2.47 ± 0.09	2.58
2	30	1	7.5	3.44 ± 0.12	3.52
3	10	1	2.5	0.92 ± 0.11	1
4	30	1	2.5	2.26 ± 0.11	2.38
5	20	3.68179	5	2.72 ± 0.13	2.72
6	20	2	5	2.83 ± 0.15	2.58
7	36.8179	2	5	3.54 ± 0.07	3.45
8	10	3	7.5	1.37 ± 0.09	1.36
9	20	2	5	2.97 ± 0.12	2.58
10	10	1	7.5	1.82 ± 0.11	1.91
11	30	3	7.5	3.29 ± 0.15	3.32
12	20	2	5	2.71 ± 0.10	2.58
13	10	3	2.5	1.47 ± 0.17	1.5
14	20	2	5	2.16 ± 0.19	2.58
15	20	2	0.795518	1.91 ± 0.16	1.82
16	20	0.318207	5	2.64 ± 0.10	2.48
17	3.18207	2	5	0.72 ± 0.13	0.6461
18	20	2	5	2.31 ± 0.16	2.58
19	30	3	2.5	3.21 ± 0.24	3.23
20	20	2	9.20448	2.74 ± 0.10	2.66

The EPS concentration (g/L) was recorded for the experiments conducted in triplicate. The association between dependent and independent variables is denoted as a second-degree regression Equation 2.


$$\begin{array}{l} Y=\beta_{0}+\beta_{1}X_{1}+\beta_{2}X_{2\;\;}+\beta_{3}X_{3}+\beta_{12}X_{1}X_{2}+\beta_{13}X_{1}X_{3}\\ +\beta_{23}X_{2}X_{3}+\beta_{11}X_{1^{2}}+\beta_{22}X_{2^{2}}+\beta_{33}X_{3^{2}} \end{array}$$ (2)

where *Y* is the dependent variable (EPS concentration), *X_1_*, *X_2_* and *X_3_* are independent variables (galactose, yeast extract, and ammonium sulfate), *β_0_* is the intercept, *β_1_*, *β_2_* and *β_3_* are linear coefficients, *β_12_*, *β_13_* and *β_23_* are the interaction coefficients and *β_11_*, *β_22_* and *β_33_* are the quadratic coefficients (25).

The response was analyzed using ANOVA to determine the *F*-value (model significance), *p*-value, lack-of-fit *F*-value, and R^2^. The optimum concentrations and interaction effects of the variables were calculated using the fitted equation, and 2D contour plots and 3D response surface plots were obtained.

The validation experiment was performed using the optimum concentrations of galactose (30 g/L), ammonium sulfate (1 g/L), and yeast extract (7.5 g/L) to verify the RSM results. The concentration of EPS (g/L) and statistical results were compared. Each experiment was carried out in triplicate, and the mean EPS production was expressed in g/L.

### 2.4 RSM-based optimization of prebiotic potential of EPS-4412 in BPJ fermentation

The optimization of the prebiotic potential of EPS-4412 through fermentation of banana pseudostem juice (BPJ) was statistically achieved using RSM, particularly central composite design (CCD). Three major factors influencing the prebiotic potential of EPS, including inoculum concentration, incubation time, and EPS concentration, were considered for CCD experimentation. Using the CCD design matrix generated by Design Expert software version 13, 20 experimental runs with six replicated center points were utilized to ascertain the three variables’ ideal concentration and interactive effect. The selected factors were analyzed at 5 different levels: high, medium, low, and axial points (−1, 0, +1, +*α*, and -α) (26). The design matrix used for CCD experimental analysis is provided in Table 3. Fermentation of BPJ without EPS was carried out as a control in triplicate.

**Table 3:** CCD design matrix for optimizing the prebiotic property of EPS-4412.

Run order	A: EPS concentration (%)	B: Inoculum size (%)	C: Incubation time (h)	Viable cell count [CFU/mL (× 10^7^)]	
				Experimental value	Predicted value
1	0.3	2	48	5.4	6.45
2	0.5	3	24	46.4	45.08
3	0.1	3	72	10.3	12.36
4	0.3	2	88.363	1.5	2.92
5	0.3	3.68179	48	33.6	37.34
6	0.5	3	72	21.5	15.90
7	0.3	0.318207	48	12.0	14.25
8	0.3	2	48	18.4	6.45
9	0.5	1	24	1.2	−5.09
10	0.636359	2	48	16.3	21.74
11	−0.03636	2	48	45.0	45.55
12	0.3	2	48	3.6	6.45
13	0.3	2	48	4.4	6.45
14	0.5	1	72	14.2	14.83
15	0.1	1	24	25.4	26.76
16	0.3	2	7.63697	15.9	20.46
17	0.1	1	72	38.0	35.08
18	0.1	3	24	58.0	53.14
19	0.3	2	48	6.0	6.45
20	0.3	2	48	1.9	6.45
No-EPS control	0	1	24	1.02	-

The banana pseudostem was washed with distilled water prior to juice extraction. After removing the outer sheath, the inner central core of the banana pseudostem (diameter < 6 cm) was cut into round slices. Using a mortar and pestle, the pieces were finely ground, and the juice was extracted through a muslin cloth. The obtained juice (20 mL) was transferred into conical flasks with different concentrations of EPS-4412, as determined by the CCD experimental design, and sterilized. After cooling, different inoculum sizes (%) of the consortium were added to 20 flasks and incubated in a rotary shaker (125 rpm) at 37 °C. The samples were withdrawn at various intervals based on the experimental design, serially diluted, and plated onto MRS agar plates.

The dependent and independent variable relationship is expressed using the second-degree regression Equation 3:


$$\begin{array}{l} Y=\beta_{0}+\beta_{1}X_{1}+\beta_{2}X_{2\;\;}+\beta_{3}X_{3}+\beta_{12}X_{1}X_{2}+\beta_{13}X_{1}X_{3}\\ +\beta_{23}X_{2}X_{3}+\beta_{11}X_{1^{2}}+\beta_{22}X_{2^{2}}+\beta_{33}X_{3^{2}} \end{array}$$ (3)

In this model, *Y* is the response (viable cell count), *X_1_*, *X_2,_* and *X_3_* denote independent variables (EPS concentration, inoculum size, and incubation period), *β_0_* corresponds to the intercept, *β_1_, β_2,_* and *β_3_* are linear coefficients, *β_12_*, *β*_*13*,_ and *β_23_* represent interaction coefficients and *β_11_*, *β_22,_* and *β_33_* are the quadratic terms.

The results of CCD-based RSM were analyzed by ANOVA to calculate the *F*-value, which determines the model’s statistical significance. Additionally, other statistical parameters, including the lack of fit F-value, R^2^ value, and *p*-value, were estimated. Three-dimensional (3D) response surface and contour images were developed using the fitted equation, which was utilized to assess the optimum concentration and interaction effects of variables (24). A validation experiment was carried out under the optimum fermentation parameters, including EPS concentration (0.1%), inoculum concentration (3%), and incubation period (24 h) to validate the RSM results, and the viable cell count was estimated.

### 2.5 Assessment of cell viability, pH, and titratable acidity

The viability of the bacterial cells was monitored by serially diluting 1 mL of fermented banana pseudostem juice (FBPJ) up to 10^6^ using sterile saline. Samples diluted (100 μL) were spread plated on MRS-agar and incubated at 37 °C for 48 h. After 30 days of FBPJ storage at 4 °C, flasks with higher colony counts were selected for further experimental studies. The viable cell count was expressed as CFU/mL.

The pH of the FBPJ supernatant was measured after centrifugation (8,000 rpm for 15 min at 4 °C). Three drops of 1% phenolphthalein were added to FBPJ and were titrated against NaOH (0.1 N) until a permanent pink color was observed (27). The titratable acidity was measured as the percentage of malic acid by weight and calculated using Equation 4.


$$\begin{array}{l} \text{Titratable acidity}\;(\%)\\ =\frac{\text{Quantity of NaOH}\;(\mathrm{mL})\times 0.0067\times 100}{\text{Quantity of sample}\;(\mathrm{mL})} \end{array}$$ (4)

### 2.6 Estimation of total carbohydrate and polyphenolic content

Using standard D-glucose and the phenol-sulfuric acid technique, the total sugar content of FBPJ upon fermentation was verified (21). The total phenolic content was determined using the Folin–Ciocalteu (FC) method with gallic acid as the standard, with slight modifications (28). FBPJ (300 μL) was mixed with 150 μL of 0.1 M FC reagent, then incubated at room temperature for 10 min. After adding 500 μL of a 7.5% Na_2_CO_3_ solution, the mixture was vortexed for 2 mins and left in the dark conditions for a further hour. The sample mixture absorbance was recorded at 765 nm to calculate the carbohydrate phenolic content in gallic acid (mg) equivalents per liter (GAE/L).

### 2.7 Volatile organic compound analysis

The presence of volatile organic compounds (VOCs) was observed using GC–MS analysis. The FBPJ supernatant (1 mL) was mixed with methanol (2 mL) to extract volatile compounds. Then, the collected samples were subjected to GC analysis (Perkin Elmer, Clarus 680) using a fused silica column packed with Elite-5MS (5% biphenyl 95% dimethylpolysiloxane, 30 m × 0.25 mm ID × 0.25 μm df). The compounds were chromatographically separated using the carrier gas, helium at a flow rate of 1 mL/min. The spectrum obtained for the known components was determined using TurboMass ver 5.4.2 and the NIST (2008) library.

### 2.8 *In vitro* compatibility test between FBPJ and probiotic bacteria

The compatibility of the FBPJ with the probiotic strains used for fermentation was assessed using the agar well diffusion assay with modifications (29). FBPJ was fermented using the probiotic consortium with EPS-4412 as the prebiotic under the optimum conditions. After 24 h, the FBPJ supernatant was obtained by centrifugation and filtered. Fresh cultures of three probiotic strains used for fermentation in MRS broth were uniformly spread on the Mueller-Hinton agar (MHA) plates. Approximately 6 mm-sized wells were created aseptically, 100 μL of the FBPJ was added, and incubated at 37 °C for 24 to 48 h. The zone of inhibition was measured in mm.

### 2.9 Biological properties of FBPJ

#### 2.9.1 Antibacterial activity

Freshly prepared cultures of pathogens such as *S. flexneri* MTCC 1457, *S. enterica* MTCC 1164, and *B. cereus* MTCC 1272, grown in Mueller-Hinton broth (MHB), were used in the agar well diffusion method. Standard ampicillin (100 μg/mL) and PBS were used as positive and negative controls, respectively. The pathogens were lawn-cultured on MHA, and 7 mm diameter wells were bored into the medium to hold 100 μL of the samples. After 24–28 h of incubation, the zone of inhibition was measured around the wells (mm) (30).

#### 2.9.2 Antioxidant activity

The antioxidant activity of the samples has been determined by centrifugation at 10000 rpm for 10 min at 4 °C. Equal parts of the sample and 0.2 mM DPPH methanolic solution were combined to determine the DPPH radical scavenging activity. The mixture was incubated at room temperature in dark conditions for 30 min, and the absorbance of the mixture was recorded at 517 nm (30).

The reducing power of EPS-4412 was determined using reactions consisting of 500 μL of the samples, 500 μL of 0.2 M sodium phosphate buffer (pH 6.6), and 500 μL of potassium ferrocyanide (1%, w/v), followed by incubating at 50 °C for 30 min. After adding 500 μL of trichloroacetic acid (10%), it was centrifuged at 4500 rpm for 10 min at 4 °C. After the removal of the upper layer, 1.5 mL of the sample was added with 300 μL of 0.1% freshly prepared ferric chloride. The Prussian blue color was developed, and the contents were measured at 700 nm using a visible spectrophotometer (xMark™ microplate absorbance spectrophotometer) (30).

The superoxide anion scavenging activity was assessed by mixing 100 μL of the sample, 50 μL of 50 mM Tris HCl buffer (pH 8.2), and 25 μL of pyrogallol (1.5 mM), followed by incubation at 25 °C for half an hour. To terminate the reaction, 10 μL of 8 mM HCl was added, and absorbance was recorded at 325 nm (31). DPPH and superoxide anion scavenging assays were conducted in comparison with ascorbic acid (range of 2–10 mg/mL) as the standard and measured using the following Equation 5.


$$\text{Scavenging activity}\;(\%)=1-\frac{\text{Absorbance of sample}}{\text{Absorbance of control}}\times 100$$ (5)

### 2.10 Statistical analysis

The results were provided as mean ± standard deviation (*n* = 3). The Origin-Pro 2025 software (Northampton, Massachusetts, United Stattes) was used for the statistical analysis.

## 3 Results

### 3.1 Optimization of EPS production

EPS-4412 extracted from *P. pentosaceus* 4412 was isolated from fermented *Manilkara zapota* juice. The molecular mass of EPS-4412 was measured to be 74 kDa and composed of mannose, glucose, and rhamnose, with a molar ratio of 90.5:3.48:1. The functional groups, such as O–H, C–H, C=O, and C–O–C, with the existence of *α*- and *β*-glycosidic linkages, were identified. EPS-4412 was further characterized as smooth, glossy, irregular, compact, stacked flaky structures, semi-crystalline, and thermally stable (19). In the present study, the EPS-4412 production has been enhanced to facilitate the exploitation of this valuable bioactive compound for future studies (process optimization).

#### 3.1.1 OFAT analysis

**Figure 1:**
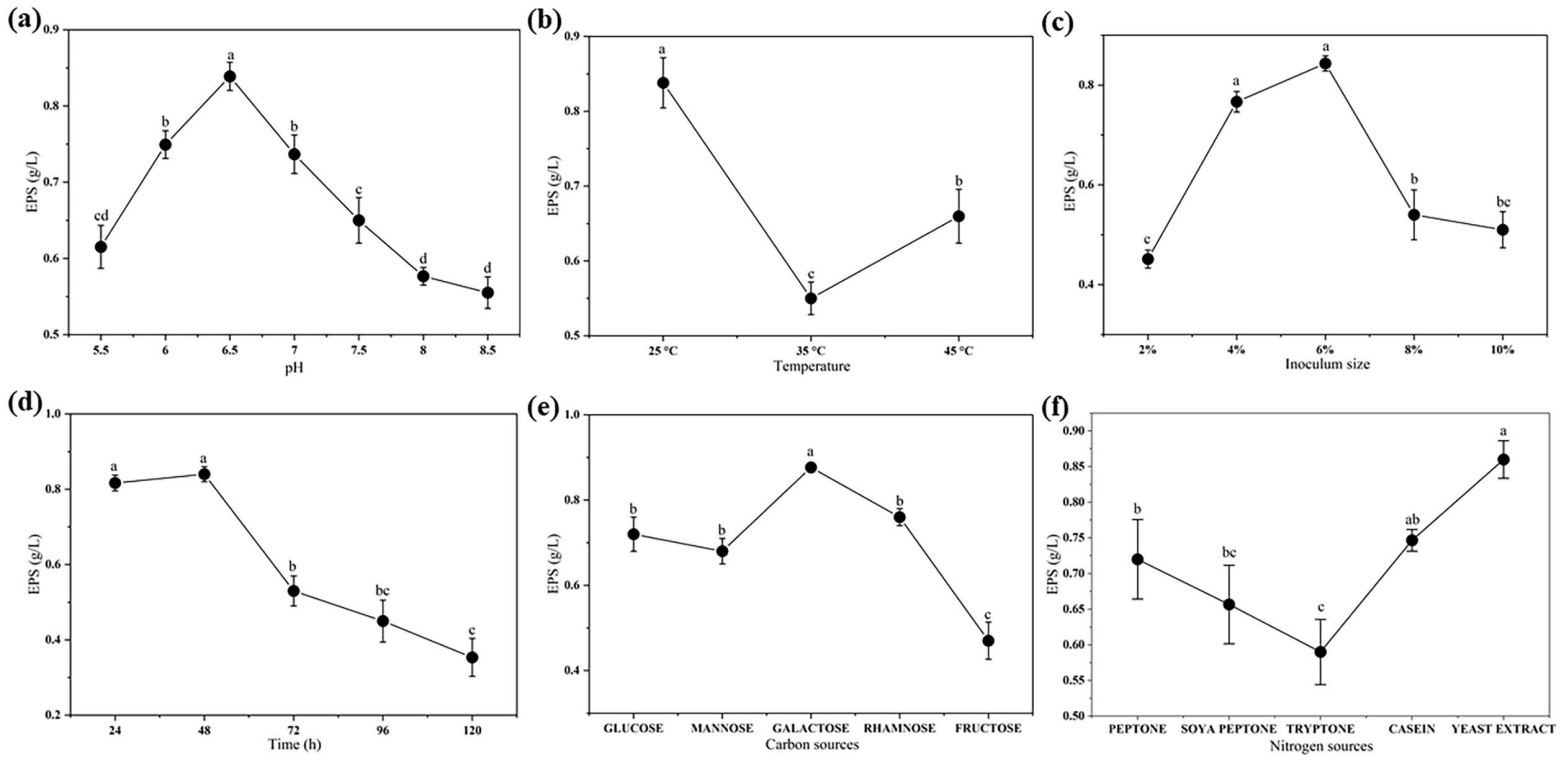
Preliminary screening of media components by OFAT analysis (mean ± SD): **(a)** Effect of different pH (5.5, 6.0, 6.5, 7.0, 7.5, 8.0, and 8.5); **(b)** effect of different temperature (25, 35, and 45 °C); **(c)** effect of different inoculum size (2, 4, 6, 8, and 10%); **(d)** effect of different incubation time (24, 48, 72, 96, and 120 h); **(e)** effect of different carbon sources (glucose, mannose, galactose, rhamnose, and fructose); and **(f)** effect of different nitrogen sources (peptone, soya peptone, tryptone, casein, and yeast extract) on EPS production by *P. pentosaceus* 4412. Different letters represent significant differences (*p* < 0.05) among variables.

Figure 1 shows the results of the OFAT experiment. Initially, the optimal pH and temperature for EPS production were determined to be pH 6.5 and 25 °C. At inoculum size and incubation period 6% and 48 h, the production of EPS-4412 was higher than at other inoculum sizes and incubation periods, respectively. In addition, the EPS concentration decreased when the incubation period exceeded 48 h. Among five carbon and nitrogen sources, galactose and yeast extract, with the maximum production of 0.88 ± 0.015 g/L and 0.86 ± 0.026 g/L, were the most suitable components for statistical optimization.

#### 3.1.2 PB analysis

The PB design was used to screen significant medium components influencing EPS production by *P. pentosaceus* 4412. Nine components (with 2 dummy factors) were screened using a 12-experiment design at two levels. Table 1 represents the response (EPS production) of the 9 variables examined. Run order 13 shows the EPS concentration of an OFAT optimized medium (1.23 ± 0.11 g/L). Run order 10 produced the most EPS (2.11 ± 0.27 g/L), whereas run order 5 produced the least (0.64 ± 0.11 g/L).

**Figure 2:**
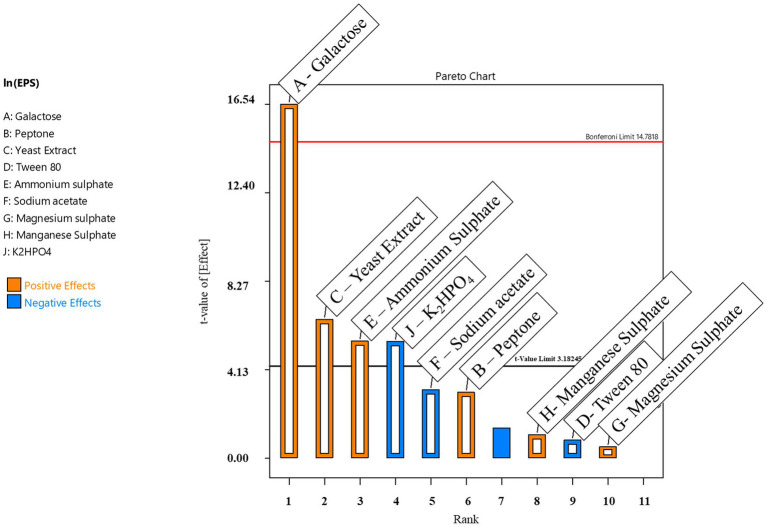
The Pareto plot shows the related levels of significance of variables on EPS production through PB analysis.

The Pareto chart depicts the primary influence of individual factors on EPS production (Figure 2). Based on the main effects, galactose, ammonium sulfate, yeast extract, magnesium sulfate, peptone, and manganese sulfate at high levels significantly influenced EPS production. In contrast, K_2_HPO_4_, sodium acetate, and Tween-80 had negative effects on EPS production. The ANOVA of the experimental model is represented in Table 4. The *F*-value of 44.14 specifies that the experimental model is statistically significant. Further, the model terms are found to be significant, as the *p*-values are less than 0.005. The results suggested that out of the nine variables examined, galactose, ammonium sulfate, and yeast extract influence greater EPS generation (Table 4).

**Table 4:** ANOVA for the experimental model in PB analysis.

Source	Sum of squares	df	Mean square	F-value	*p-*value	Significance
Model	1.59	9	0.1766	44.14	0.0223*	significant
A-Galactose	1.09	1	1.09	273.53	0.0036*	
B-Peptone	0.0379	1	0.0379	9.48	0.0912	
C-Yeast Extract	0.1683	1	0.1683	42.07	0.0230*	
D-Tween 80	0.0029	1	0.0029	0.7155	0.4867	
E-Ammonium sulfate	0.1196	1	0.1196	29.91	0.0318*	
F-Sodium acetate	0.0408	1	0.0408	10.21	0.0855	
G-Magnesium sulfate	0.0011	1	0.0011	0.2844	0.6471	
H-Manganese sulfate	0.0049	1	0.0049	1.22	0.3852	
J-K_2_HPO_4_	0.1192	1	0.1192	29.80	0.0320*	
Residual	0.0080	2	0.0040			
Cor total	1.60	11				

R^2^: 0.9950; ^*^significant at *p* ≤ 0.05.

#### 3.1.3 EPS production optimization through RSM

To maximize EPS production, the concentration and interaction of significant variables, such as galactose, ammonium sulfate, and yeast extract, were optimized using CCD-based RSM analysis. Table 2 presents the predicted and experimental values of EPS secretion (g/L) obtained from 20 experimental runs. The run order 7 showed the maximum EPS production with an experimental value of 3.54 ± 0.07 g/L and a predicted value of 3.45 g/L. The run order 17 exhibited the lowest EPS production with an experimental value of 0.72 ± 0.13 g/L and a predicted value of 0.64 g/L. The second-order polynomial equation, based on the significant factors and their interactions, is given in the following Equation 6:


$$\begin{array}{l} Y=+2.58+0.8342\;A+0.0736\;B+0.2496+0.0888\;\mathrm{AB}\\ +0.0588\;\mathrm{AC}-0.2638\;\mathrm{BC}-0.1882\;A^{2}+0.0063\;B^{2}\\ -0.1210\;C^{2} \end{array}$$ (6)

where *Y* is the response (EPS concentration) and *A*, *B,* and *C* denote galactose, ammonium sulfate, and yeast extract concentrations, respectively.

Table 5 represents the ANOVA of the multiple regression model for EPS production. The *F-*value and *p*-value of the model were 22.47 and 0.0001, respectively, which indicates that the model is statistically significant. The probability (0.01%) of the model *F-*value designates the possibility of noise. If the *p-*value is below 0.05, the model has a significant effect, whereas a *p*-value greater than 0.2 indicates that he model is insignificant. The significant model terms with notable influence on optimization include the linear coefficient of galactose (A; *p* < 0.0001), yeast extract (C; *p* < 0.05), the interactive coefficient, BC (*p* < 0.05), and quadratic coefficients, A^2^ (*p* < 0.05). The *F*-value of 0.9563 denotes a non-significant lack of fit, reflecting that the experimental results are appropriate and fit the model well. The model was effective since the R^2^ value showed a good correlation between the actual and predicted values. As the R2 value ranges between 0 and 1.0, a value approaching 1.0 indicates a better-fitting statistical model. The R^2^ value of 0.9529 indicated that the model is statistically fit, with 95.29% of the variability in the response can be explained by the current model. The projected R^2^ value of 0.8872 is in reasonable agreement with the corrected R^2^ value of 0.9105, with a difference of less than 0.2. Adequate accuracy, which assesses the signal-to-noise ratio, was more than 4, indicating a solid model. An adequate precision ratio of 16.871 was found, indicating that the model is acceptable.

**Table 5:** ANOVA of the multiple regression model for EPS production.

Source	Sum of squares	df	Mean square	F-value	*p*-value	Significance
Model	11.76	9	1.31	22.47	< 0.0001**	significant
A-Galactose	9.50	1	9.50	163.48	< 0.0001**	
B-Ammonium sulfate	0.0739	1	0.0739	1.27	0.2859	
C-Yeast extract	0.8510	1	0.8510	14.64	0.0033*	
AB	0.0630	1	0.0630	1.08	0.3223	
AC	0.0276	1	0.0276	0.4750	0.5064	
BC	0.5565	1	0.5565	9.57	0.0114*	
A^2^	0.5102	1	0.5102	8.78	0.0142	
B^2^	0.0006	1	0.0006	0.0098	0.9230	
C^2^	0.2109	1	0.2109	3.63	0.0859	
Residual	0.5813	10	0.0581			
Lack of Fit	0.0906	5	0.0181	0.1846	0.9563	not significant
Pure Error	0.4907	5	0.0981			
Cor Total	12.34	19				

R^2^: 0.9529, Adjusted R^2^: 0.9105, C. V. %: 10.15, Adeq Precision: 16.8710, **Significant at *p* ≤ 0.001, *Significant at *p* ≤ 0.05.

**Figure 3:**
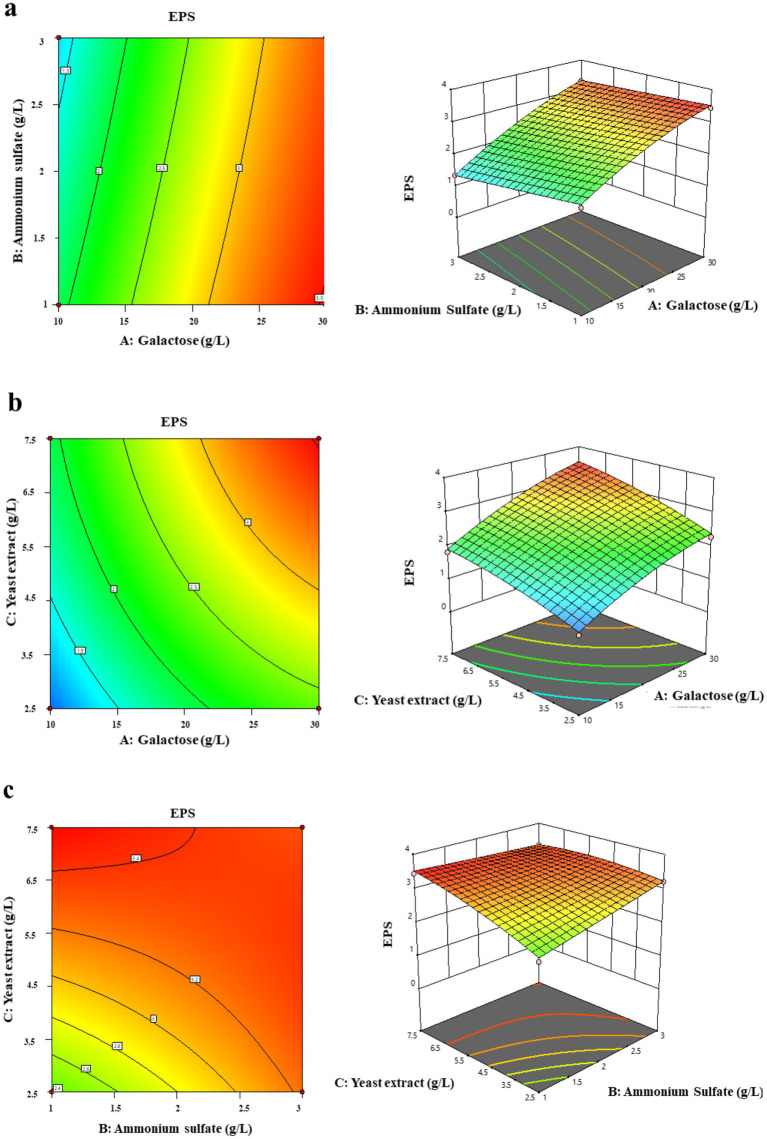
Surface plot and response surface plots of EPS production optimization showing interactions between independent variables: **(a)** Galactose and ammonium sulfate, **(b)** galactose and yeast extract, **(c)** ammonium sulfate and yeast extract.

The ANOVA results are enclosed with three linear terms (A, B, C), three quadratic terms (A^2^, B^2^, C^2^), three interaction terms (AB, AC, BC), and one block term. Notably, the linear effects of galactose and yeast extract were substantially greater than their respective quadratic effects. Galactose has the strongest linear influence compared to the other factors. The association between ammonium sulfate and yeast extract was more pronounced than the other relationships. These response surface graphs were created using the concentrations of two variables, with the third variable kept at its optimal level. These graphs show the interacting impacts of factors and assist estimate the optimal concentration of each variable for maximal EPS generation. Elliptical or saddle-shaped contour plots demonstrate considerable interaction between factors. Figure 3a represents the interaction of galactose and ammonium sulfate, whereas Figure 3b depicts the interaction of galactose and yeast extract. Figure 3c represents the interaction between yeast extract and ammonium sulfate. The response surface plots exhibited an approximately saddle-shaped curvature, confirming interactions among the selected three variables. The results showed that the highest production of EPS occurs at the highest value of galactose, and a mid-value of ammonium sulfate and yeast extract.

The validation experiment was conducted, which exhibited the maximum production of EPS-4412. Galactose (30 g/L), ammonium sulfate (1 g/L), and yeast extract (7.5 g/L) were supplemented as the major medium components, with a pH of 6.5. The optimized medium was cultured with 6% (v/v) inoculum, followed by incubation for 48 h at 25 °C.

Under the optimized conditions, the validation experiment resulted in EPS production of 3.46 ± 0.16 g/L. Compared to unoptimized conditions of 0.62 ± 0.12 g/L, medium optimization resulted in a 5.5-fold increase in EPS production.

### 3.2 RSM-based optimization of prebiotic potential of EPS-4412 in BPJ fermentation

To optimize the prebiotic potential of EPS-4412 assessed in terms of viable cell count, five levels of EPS concentration, inoculum concentration, and incubation period were analyzed to estimate the optimum parameters and interactive effects of these variables. The experimental and predicted viable cell count from CCD-based RSM are represented in Table 3. Run order 18 exhibited the highest prebiotic potential in the experiment and predicted viable cell count of 58 × 10^7^ and 53.14 × 10^7^ CFU/mL, respectively. Run order 9 exhibited the lowest prebiotic potential with the experimental and predicted values of 1.2 × 10^7^ and −5.09 × 10^7^ CFU/mL, respectively. Also, the control experiment without EPS yielded much lower viable cell count of 1.02 × 10^7^ CFU/mL, providing evidence for the role of EPS-4412 in enhancing the probiotic growth. These results were analyzed by performing multiple regression analysis and ANOVA. The second-order polynomial equation, based on the significant factors and their interactions, is given in the following Equation 7:


$$\begin{array}{l} Y=+6.45-7.08\;A+6.86\;B-5.21\;C+5.95\;\mathrm{AB}\\ +2.90\;\mathrm{AC}-12.28\;\mathrm{BC}+9.62\;A2+6.84\;B2+1.86\; \end{array}$$ (7)

Where *Y* is the response (viable cell count) and *A*, *B,* and *C* denote EPS concentration, inoculum concentration, and incubation period, respectively.

The ANOVA for the response model for the prebiotic potential of EPS-4412 is represented in Table 6. The model *F*-value of 15.71 and *p*-value of <0.0001 implied that the model is highly significant, and it is indicated that there is only a 0.01% chance that noise would be the reason for an *F*-value this large. Model terms with a significant *p-*value, i.e., less than 0.05, and insignificant *p*-value, i.e., higher than 0.1, were estimated. In the prebiotic potential optimization by CCD-based RSM, the linear coefficients of A, B, and C, interactive coefficients AB and BC, and quadratic coefficients A^2^ and B^2^ (*p* < 0.05) are the significant terms with a notable effect on the prebiotic potential of EPS-4412, thus enhancing the viable cell count of FBPJ.

**Table 6:** Analysis of variance (ANOVA) for the response surface model for prebiotic potential.

Source	Sum of squares	df	Mean square	F-value	*p*-value	Significance
Model	5093.63	9	565.96	15.71	< 0.0001***	significant
A-EPS conc.	684.24	1	684.24	18.99	0.0014**	
B-Inoculum size	643.24	1	643.24	17.86	0.0018**	
C-Incubation time	371.39	1	371.39	10.31	0.0093*	
AB	283.22	1	283.22	7.86	0.0187*	
AC	67.28	1	67.28	1.87	0.2017	
BC	1205.41	1	1205.41	33.46	0.0002***	
A^2^	1332.61	1	1332.61	36.99	0.0001***	
B^2^	674.39	1	674.39	18.72	0.0015**	
C^2^	49.62	1	49.62	1.38	0.2677	
Residual	360.22	10	36.02			
Lack of Fit	183.26	5	36.65	1.04	0.4852	not significant
Pure Error	176.97	5	35.39			
Cor Total	5453.85	19				

R^2^: 0.9340, Adjusted R^2^: 0.8745, C. V. %: 31.67, Adeq Precision: 13.7213, ***Significant at *p* ≤ 0.001, **Significant at *p* ≤ 0.005, *Significant at *p* ≤ 0.05.

The experimental responses fitted the model as the lack of fit *F*-value (0.4852) was not significant. The R^2^ value of 0.9340 indicated that the model is a good fit, and 93.40 of % variability can be explained by the present model. Also, the predicted R^2^ value of 0.6808 is in reasonable agreement with the adjusted R^2^ value of 0.8745, as the difference was less than the ideal value of 0.2. The model’s adequate precision value of 13.7213 indicated an adequate signal-to-noise ratio. The lower CV value of 31.67% indicated the credibility of the statistical experimentation.

**Figure 4:**
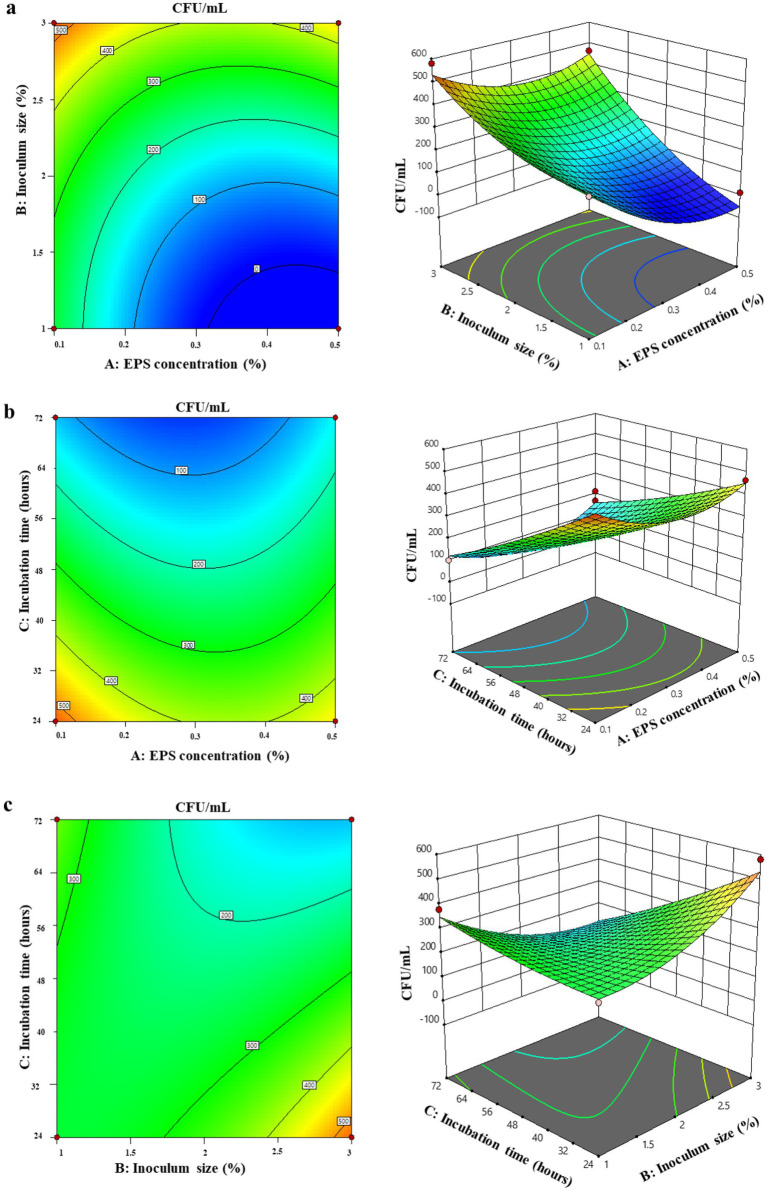
Surface plot and response surface plots of prebiotic potential optimization showing interactions between independent variables: **(a)** EPS concentration and inoculum size, **(b)** EPS concentration and incubation time, **(c)** inoculum size and incubation time.

ANOVA table (Table 6) indicated that the quadratic term of A^2^ was much increased in comparison with its linear and interactive effects. The linear effect of EPS concentration was more influential in the prebiotic property compared to the other linear effects. On analyzing the interactive effects, BC exhibited a significantly higher effect in comparison with other interaction effects. Similar results were observed with contour and response surface plots. 3D response and 2D contour plots indicated interactions between the selected independent variables on the prebiotic property of EPS in FBPJ. Figure 4a shows the interactions between EPS concentration and inoculum size on prebiotic potential. The parallel curved contour plot and circular response surface plot indicated that the interaction between EPS concentration and inoculum size concerning prebiotic potential is insignificant. Both the highest and lowest concentrations of EPS exhibited higher probiotic viable cell count, but not in the medium concentration. Specifically, the lowest viable counts were consistently observed at the mid-range EPS level (0.3%) when combined with the intermediate inoculum size (2%) and incubation time (48 h), as reflected in several center-point runs (run orders 1, 8, 12, 13, 19 and 20). In contrast, higher viable counts were obtained when low (0.1%) or high (0.5%) EPS concentrations were combined with a higher inoculum level and a shorter incubation time, particularly in run orders 18 and 2.

Figure 4b represents the interaction between EPS concentration and incubation time on prebiotic potential. Circular contour and response surface plots represented no significant interaction between the two variables. Lower incubation time was optimum for the prebiotic potential, as the highest viable cell count was observed after 24 h of incubation. Similar to the previous RSM plot (Figure 4a), EPS exhibited a significant effect at both higher and lower concentrations. Figure 4c demonstrates the interaction between inoculum concentration and incubation time on prebiotic potential. Saddle-shaped plots indicated moderate interaction between the selected variables.

For validation, the optimum combination of 0.1% EPS, 3% inoculum, and 24 h incubation (which matched the run order 18) was experimentally tested, yielding experimental and predicted values of 56.9 × 10^7^ and 53.14 × 10^7^ CFU/mL, respectively, demonstrating optimal fermentation conditions for increased prebiotic stimulant potential. These findings show that EPS supplementation positively influences the probiotic viability; however, the efficacy depends on inoculum concentration and fermentation time, as evidenced by the interaction pattern observed in the response surface plots. The interaction-dependent behavior highlights the importance of optimized process conditions for maximizing the prebiotic stimulant potential.

### 3.3 Cell viability, pH, and titratable acidity

**Figure 5:**
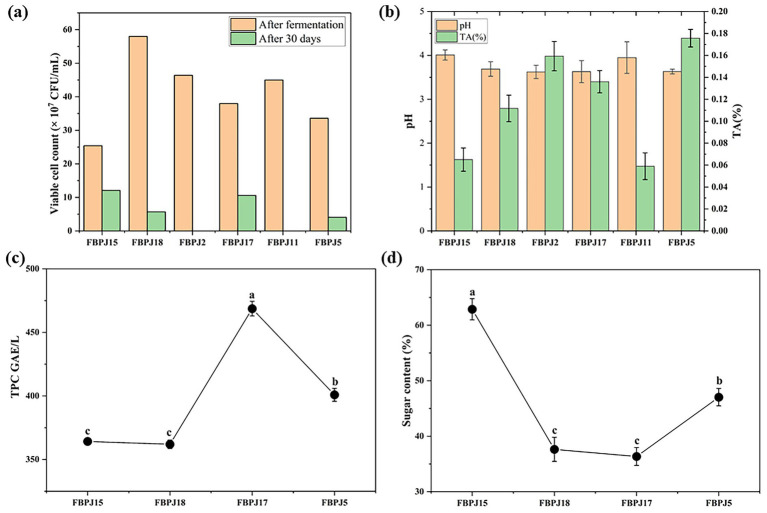
Determination of **(a)** Viable cell counts, **(b)** pH value and titratable acidity (TA%), **(c)** sugar content, and **(d)** TPC in FBPJ samples. Significant differences (*p* < 0.05) among samples are represented by the lowercase letters.

Each sample was plated based on its incubation duration, and the six samples with the highest colonies were selected. Figure 5a depicts the viable cell counts after immediate fermentation and after 30 days of storage. After 24 h, the samples FBPJ15, FBPJ18, FBPJ2, FBPJ17, FBPJ11, and FBPJ5 showed increased colony counts, with log CFU/mL values of 8.4, 8.76, 8.67, 8.58, 8.65, and 8.53, respectively. After 30 days, a colony count was performed on these samples to verify viability. Samples FBPJ2 and FBPJ11 exhibited no colonies; the other four samples had viable colony counts. However, a decrease in colony number was seen in all four samples, most likely due to competition for nutrients and acid production by mixed LAB fermentation (15). FBPJ17’s viability was maintained after 30 days of refrigeration, with a log CFU/mL of 8.03, compared to the other three samples. During storage, samples with greater colony counts and acid concentration reduced LAB growth and output, thus halting the fermentation process. Acidification of FBPJ was validated by measuring the pH values of FBPJ18 and FBPJ17, which were found to be 3.69 ± 0.16 and 3.63 ± 0.25, respectively. Figure 5b shows the variations in pH and titratable acidity (%) of fermented samples. These results show a slight decrease compared to FBPJ15 (pH 4.01 ± 0.11) and FBPJ5 (pH 3.63 ± 0.25). The TA of FBPJ15, FBPJ18, FBPJ2, FBPJ17, FBPJ11, and FBPJ5 were observed to be 0.065 ± 0.011%, 0.112 ± 0.012%, 0.159 ± 0.013%, 0.136 ± 0.010%, 0.059 ± 0.012%, and 0.176 ± 0.008%, respectively.

### 3.4 Estimation of total carbohydrate and total polyphenolic content

The sugar content of the FBPJ18 and FBPJ17 was calculated to be 37.63 ± 2.17% and 36.35 ± 1.61%, respectively (Figure 5c). The phenolic content of all four samples increased after fermentation for 24 h, 48 h, and 72 h, respectively. In particular, the TPC of FBPJ17 was 470.89 ± 5.09 GAE/L when incubated for 72 h with an inoculum size of 1% and an EPS concentration of 0.1%. The TPC content of FBPJ15, FBPJ18, FBPJ5, and control was determined as 364.22 ± 1.92, 359.78 ± 5.22, 462 ± 3.33, and 45.33 ± 2.54 GAE/L which are lower than FBPJ17 (Figure 5d).

### 3.5 Volatile compounds analysis

**Figure 6:**
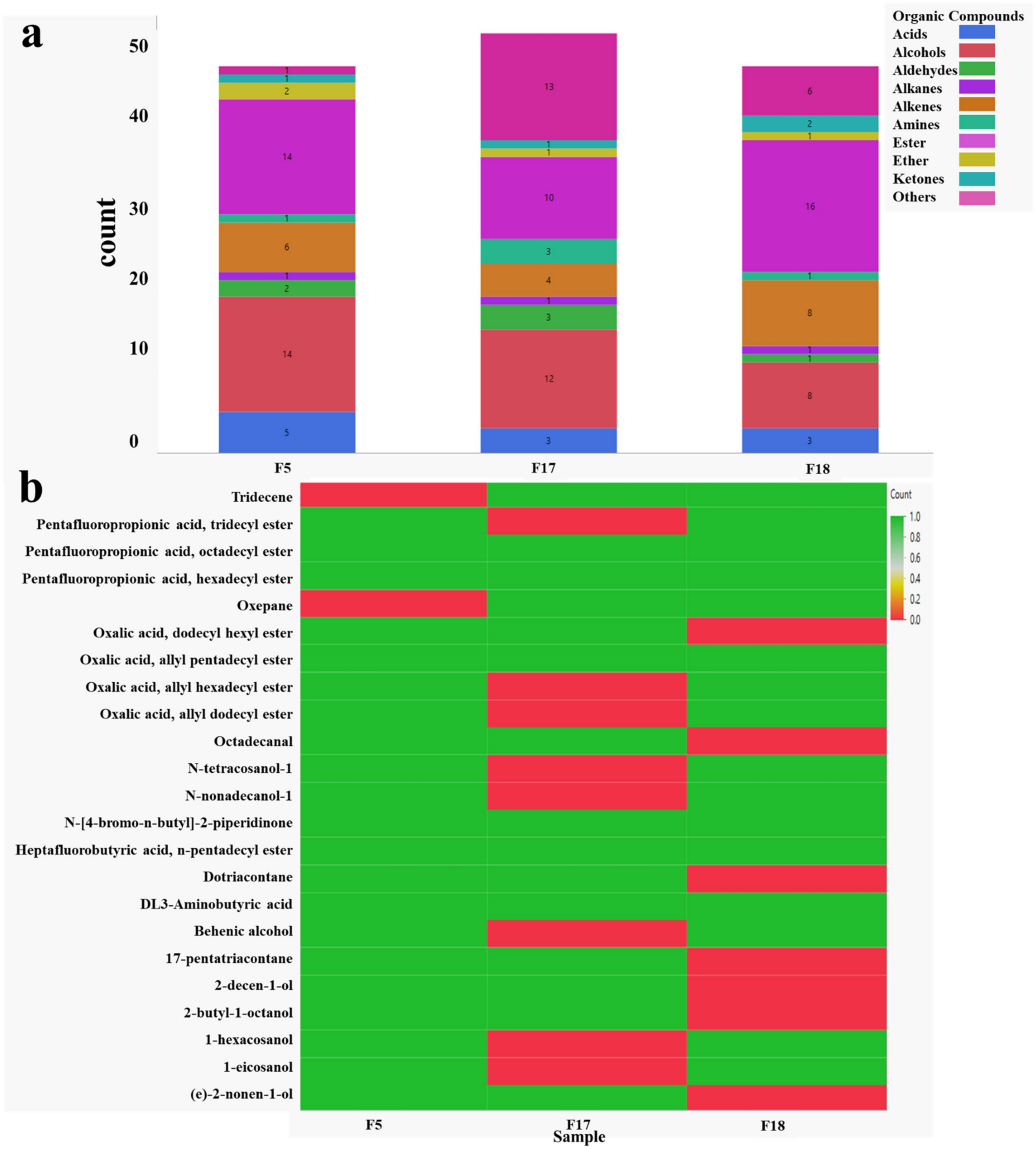
**(a)** Comparative analysis of the number of each volatile compound present in FBPJ and **(b)** heat map of common volatile compounds among FBPJ18, FBPJ17, and FBPJ5 samples, respectively.

The extraction and identification of VOCs in FBPJ were performed by GC–MS analysis. Comparison of aromatic volatile compounds present in FBPJ samples and their biological properties is given in Table 7. A total of 19, 45, 52, and 50 volatile compounds were found in FBPJ15, FBPJ18, FBPJ17, and FBPJ5, which comprise acids, alcohols, aldehydes, alkanes, alkenes, amines, esters, ethers, ketones, and other compounds (Figure 6a). A few notable organic compounds associated with biological properties and flavor were commonly observed in all the samples (Figure 6b).

**Table 7:** Comparison of aromatic volatile compounds present in FBPJ samples characterized by GC–MS analysis, along with their biological properties.

S. No	Volatile compounds	FBPJ15	FBPJ18	FBPJ17	FBPJ5	Flavor description	Biological properties	References
Acids								
1	DL-3-aminobutyric acid		✓	✓	✓	-	Antipathogenic	(86)
2	Oleic acid				✓	Beany or off-flavor	Lowers cholesterol and inflammation	(36)
3	2-oxo-propanoic acid				✓	-	Antimicrobial and antidiabetic activities	(87)
Alcohols								
4	3-buten-2-ol	✓				Fruity or floral aroma	-	(88)
5	N-nonadecanol-1		✓		✓	Sensory profile/spoilage marker	-	(46)
6	1-eicosanol		✓		✓	Waxy, straight-chain fatty alcohol	Antimicrobial and antioxidant	(89)
7	4-amino-1-pentanol		✓				Fruity, flavor	(10)
8	2-cyclopentylethanol		✓				Fruity, flavor, or slightly earthy or spicy	(44)
9	E-2-tetradecen-1-ol			✓		Anti-inflammatory, antihepatotoxicity, and anti-cancer	Floral or fruity aroma	(82)
10	2-decen-1-ol			✓	✓	-	Fatty rosy aroma	(36)
11	(e)-2-nonen-1-ol			✓	✓	-	Floral, fruity, and sweet aroma	(80)
12	11-tridecen-1-ol			✓		Anti-bacterial, anticancer, and antioxidant	Mild, slightly waxy aroma	(39)
13	2-hexyl-1-decanol			✓		Potential antifungal and antioxidant properties	Citrus-like flavor	(39)
14	2-butyl-1-octanol			✓	✓	-	Fruity and floral aroma	(33)
15	2-propyl-1-heptanol			✓		-	Significantly impacts the flavor profile of fermented foods.	(90)
16	(e)-2-tridecen-1-ol,				✓	-	Subtle waxy or floral aroma	(39)
17	4-dodecanol				✓	-	Earthy, soapy, waxy, and fatty odor	(42)
Aldehydes								
18	Hexadecanal			✓			Improvement of organoleptic attributes	(39)
19	Undecanal			✓			Fragrance and flavor ingredients	(35)
20	Octadecanal			✓			Pleasant, waxy or fatty odors	(39)
21	5,5-dimethyl-hexanal	✓				Anti-microbial and antioxidant	Grassy and beany flavor	(36)
22	9-octadecenal	✓				Antimicrobial	Off-flavor and unpleasant taste	(41)
Ethers								
23	Vinyl lauryl ether				✓			(44)
24	1-(ethenyloxy)-octadecane, / octadecyl vinyl ether				✓		Sensory characteristics	(35)
Esters								
25	Oxalic acid, heptyl propyl ester	✓				Antioxidant	Faint fruity aroma	(37)
26	Propionic acid, 1-methylethyl ester/ethyl isobutyrate	✓				-	Fruity, sweet, and floral aroma	(91)
27	Sulfurous acid, 2-propyl heptyl ester	✓				-	Sulfur-like, green, or even honey-like notes	(88)
28	Propanoic acid, ethenyl ester	✓					Fruity aroma	(92)
29	Oxalic acid, allyl pentadecyl ester		✓	✓	✓	Anti-pest, antimicrobial, termiticidal, and maggoticidal activities	Sour, acidic flavor	(37)
30	Octadecanoic acid, 2-oxo-, methyl ester/ stearic acid methyl ester			✓		Antioxidant, anti-inflammatory, and even antiviral properties	Subtle, oily, or fatty aroma	(43)
31	Hexadecanoic acid, 2-oxo-, methyl ester			✓		Antimicrobial, antioxidant, nematicidal, and pesticidal activities	Fruity or oily	(87)
32	Oxalic acid, dodecyl hexyl ester			✓	✓	-	Floral fruity	(88)
33	Oxalic acid, propyl tridecyl ester			✓		Antioxidant, anti-inflammatory, and antimicrobial properties.	Floral fruity	(88)
Hydrocarbons								
34	3-ethyl-2,5-dimethyl-hexane	✓					Mild, slightly oily or hydrocarbon-like aroma	(33)
35	2,3,6-trimethyl-heptane	✓					Sweet-corn-like aroma	(93)
36	6-methyl-undecane	✓					Overall aroma	(33)
37	Vinylcyclopentane		✓				Pleasant, sweet, floral, and slightly fruity aroma	(33)
38	2-methyl-pentadecane,		✓				Subtle/nuanced flavor	(83)
39	5,6-dimethyl-undecane		✓				Overall aroma	-
40	1-tridecene		✓				Coconut-like or buttery.	(39)
41	Dotriacontane			✓	✓	Antimicrobial, antibacterial, and antispasmodic properties	-	(81)
42	(z)-4-tetradecene			✓		Antioxidant and antimicrobial	Aroma profile	(82)
43	(z)-4-tridecene,			✓		Antibacterial	Aroma	(39)
44	1,3(z),13-tetradecatriene			✓		-	Aroma	(39)
45	17-pentatriacontene			✓	✓		Influence the production of aroma compounds	-
Ketones								
46	2,5-dimethyl-4-hydroxy-3(2 h)-furanone	✓				Antimicrobial and potential anti-infective effects	Aroma compound	(35)
47	4,4-dimethyl-2-pentanone	✓				-	Flavor and sensory attributes	(91)
48	1-(acetyloxy)-2-butanone	✓				-	Creamy and coffee	(80)
49	2-methyl-3-pentanone	✓					unique flavor and aroma	(91)
50	2,3-pentanedione	✓				-	butterscotch, nutty, or caramel-like flavor	(94)
51	3-ethenyl-cyclohexanone,		✓			-	Fruity, green, and floral aroma.	(95)
52	2,5-dimethyl-cyclopentanone				✓	-	Nutty, buttery, and sweet aroma	(45)

Organic acids are essential metabolites that play an incredible role in fermentation, such as increasing acidity, inhibiting pathogens, and improving the shelf life of functional foods (28). LAB produce these acids through metabolizing fats, amino acids, and carbohydrates (32). The organic acids in lower content are due to the transformation into esters, alcohols, aldehydes, hydrocarbons, or other secondary metabolites (33). N-methyl-D-aspartic acid was detected in FBPJ18 to have umami taste (34), and pentadecanoic acid expressed a slightly waxy or fatty aroma in FBPJ5 (35).

Aldehydes are naturally derived by auto-oxidation of free fatty acids via mixed-strain fermentation, imparting fruity, malty, and cheesy flavors (36, 37). FBPJ15 produces 5,5-dimethyl-hexanal by the non-enzymatic browning of the Maillard process between reducing sugars and amino acids (36). The conversion of aldehydes to acids and alcohol by microbial consortium reduces the grassy flavor while enhancing fruity and pleasant aroma (11, 38). Interestingly, hexadecanal, undecanal, and octadecanal are fatty aldehydes formed in FPBJ5 that improve the organoleptic properties such as taste, texture, and fragrance (39). The compounds, including 3-heptadecenal, 5,5-dimethyl-hexanal, and 9-octadecenal, produced in FBPJ15 are observed to have grassy or beany flavor, which contributes to the overall flavor (40). These findings align with the VOC results of the mushroom-based meat analog, presenting an off-odor (41). In addition, these off-flavor compounds are reduced in samples such as FBPJ18, FBPJ17, and FBPJ5, a parallel outcome to that observed in fermented goji juice using multiple bacterial strains (32).

Alcohols are volatile chemicals formed at a low threshold value by the oxidation and degradation of polyunsaturated fatty acids, ketones, and amino acids. It enhances the sweetness and mellowness of the fermented juices (12, 40). Alcohols are generated from the degradation of sugar molecules and the catabolism of amino acids (42). Fatty alcohols such as 2-propyl-1-heptanol and 2-butyl-1-octanol have been developed in FBPJ17 and FBPJ5, encouraging fruity, floral, and mushroom flavor (33). In FBPJ5, more fatty alcohols such as (e)-2-nonen-1-ol, Trans-2-undecen-1-ol, 2-decen-1-ol, and 2-propyl-1-heptanol have contributed to floral, fruity, and rosy aroma when compared to other fermented samples (36, 43). Importantly, 2-cyclopentylethanol was identified in FBPJ18 to bestow a fruity flavor notes. This essential ethanol was absent in the fermentation study conducted by Pan et al. (12).

Esters are generated during fermentation by the enzymatic catalysis of microorganisms and non-enzymatic esterification of organic acids and alcohols (36, 40). Esters like 1-propionylethyl acetate, tetradecyl trifluoroacetate, and nonyl trifluoroacetate have a pleasant fruity and floral flavor, which is more essential than ethyl esters. In addition, they greatly impact the sensory characteristics of fermented food samples (11). FBPJ3, FBPJ5, and FBPJ12 have high levels of oxalic acid esters. In particular, these esters exhibit fruity, fatty, and oily mouthfeel, accompanied by antimicrobial and antioxidant activities. In particular, dodecanoic acid, ethenyl ester, aspartic acid methyl ester, and hexadecanoic acid, 2-oxo-, methyl ester, impart fruity and flowery notes (33).

Ketones are flavor enhancers obtained from thermal oxidation of saturated fatty acids that include 2,5-dimethyl-cyclopentanone, which imparts a fruity, buttery, and sweet aroma (44, 45). The compound, 1-(acetyloxy)-2-butanone/ acetoin, resembles a creamy taste and aroma due to its higher concentration (40, 43). Acetoin is generated through citric metabolism and is formed from 2,3-butanedione by the action of diacetyl reductase (32). Hydrocarbons are produced due to lipid oxidation associated with grassy, fatty, and soapy flavors (46).

Intriguingly, naturally occurring alkanes belonging to hydrocarbons like 1-(ethenyloxy)-octadecane and 2-methyl-pentadecane have subtle or nuanced flavor. In addition, the VOCs such as 6-methyl-undecane, 2-methyl-3-pentanone, Trans-2-undecen-1-ol, and oxalic acid, allyl nonyl ester have contributed to the overall flavor and aroma of FBPJ.

### 3.6 *In vitro* compatibility test between FBPJ and probiotic bacteria

After the incubation, no clear zone of inhibition was observed against the selected three probiotic strains. All the probiotic bacteria exhibited uniform and confluent growth in the presence of FBPJ. EPS prebiotic-mediated fermentation of BPJ showed no inhibitory activity on the growth of the probiotic strains, indicating compatibility between FBPJ and probiotic bacteria.

### 3.7 Biological properties of FBPJ

#### 3.7.1 Antibacterial activity

**Figure 7:**
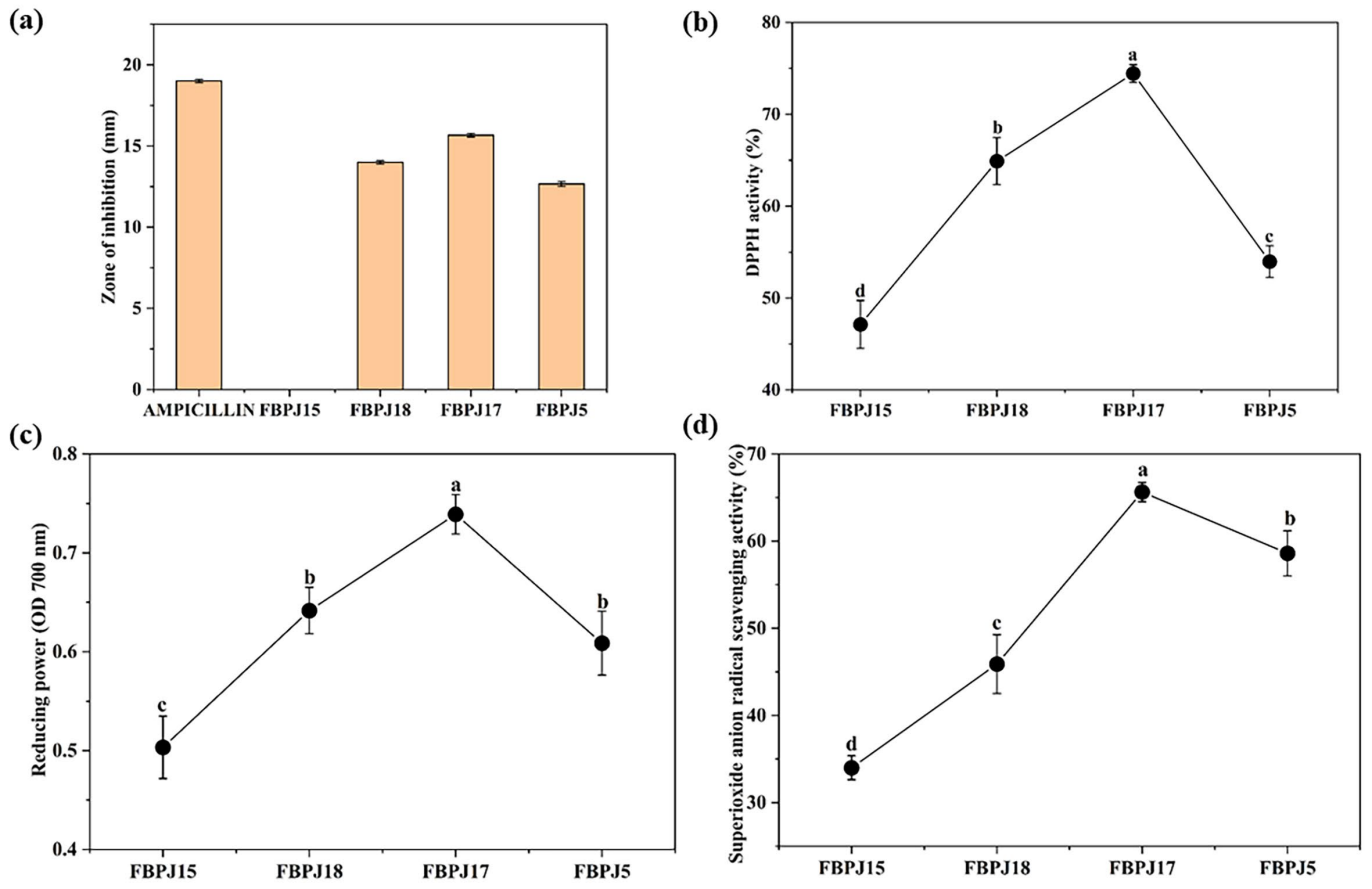
Antibacterial **(a)** and antioxidant activities of banana pseudostem juice fermented by LAB consortium **(b)** DPPH, **(c)** reducing power, and **(d)** superoxide radical scavenging potential. Significant differences (*p* < 0.05) among samples are represented by lowercase letters.

Figure 7a shows the antibacterial activity of fermented samples against *S. enterica* MTCC 1164. The growth of other pathogens, like *S. flexneri* MTCC 1457, and *B. cereus* MTCC 1272 remain unaffected by FBPJ samples. The maximum antibacterial activity was measured from FBPJ17 with a zone of inhibition of 15.7 ± 0.12 mm compared to other fermented samples. No antibacterial activity was observed for FBPJ15, but FBPJ18 (14 ± 0.1 mm) and FBPJ5 (12.7 ± 0.15 mm) inhibited the growth of *S. enterica* MTCC 1164.

#### 3.7.2 Antioxidant activity

Antioxidant activity of fermented samples is shown in Figures 7b–d. The DPPH activity of FBPJ17, FBPJ15, FBPJ18, and FBPJ5 was reported as 74.43 ± 0.96%, 47.13 ± 2.60%, 64.9 ± 2.55%, and 53.97 ± 1.72%. The reducing power of FBPJ17 was 0.72 ± 0.058, which was higher than that of other fermented samples. Among the four fermented samples, FBPJ17 and FBPJ5 revealed greater superoxide radical scavenging activity of 65.6 ± 1.1% and 58.6 ± 2.59%, respectively.

## 4 Discussion

*P. pentosaceus*, a widely recognized bacterium, produces potential EPS, and its optimization is critical to promote industrial scale-up and maximize its biological usefulness. The emerging demand for functional foods has ameliorated the need for extensive research on bioactive molecule development, raw material sustainability, and optimization of bioprocessing strategies. Recent studies have highlighted the industrial significance of microbial EPS as a multifunctional biopolymer (47). On the other hand, banana-derived substrates such as green banana flour and starch have been exploited as functional food ingredients in pasta and instant noodles owing to their nutritional value (48, 49). Based on this recent research, the current study integrates *P. pentosaceus* EPS into probiotic-based fermentation of banana pseudostem juice, an under-utilized agro-byproduct. Using RSM analysis, the fermentation parameters were statistically optimized for probiotic growth. This integrative approach enhances the functional properties of fermented beverages besides contributing to sustainable bioprocessing.

To maximize EPS production, the traditional OFAT approach was employed to evaluate essential growth parameters, such as temperature, pH, fermentation duration, inoculum size, and carbon and nitrogen sources. *L. plantarum* SP8, *L. lactis* L2, and *L. kefiranofaciens* ZW3 have also been shown to boost the generation of EPS from glucose, sucrose, and lactose (31, 50, 51), respectively. OFAT-based optimization of production resulted in 74.81 mg/L of EPS from *Bacillus amyloliquefacien*s BDIFST240014 under the optimized conditions of 37 °C for 48 h (47). Moreover, other nitrogen sources have been reported to augment EPS production, such as tryptophan (50), peptone (96), and ammonium nitrate (52). Furthermore, the culture parameters of *P. pentosaceus* 4412, such as acidic pH, inoculum size, and temperature, were similar to those related to EPS generation in *Weissella confusa* XG-3 (31). As the backbone component of microbial EPS, carbon supply is critical for its biosynthesis and substantially impacts production (53). Furthermore, galactose and yeast extract were used as the sole carbon source to enhance EPS synthesis, as described in the statistical optimization of EPS isolated from probiotic *Streptomyces griseorubens* GD5 (54).

Galactose, yeast extract, ammonium sulfate, and K_2_HPO_4_ were the significant factors resulting from PB screening. In agreement with previous reports, the production of EPS-4412 entails UDP-galactose 4-epimerase and utilizes galactose as the preferred carbon source (55). The influence of galactose on EPS-4412 production may involve both central carbon metabolism as well as monosaccharide composition, which consists of mannose, glucose, and rhamnose. Galactose is extensively metabolized by various LAB strains, including *P. pentosaceus,* through the Leloir pathway, following uptake via the phosphotransferase transport system (PTS) or permease system (56, 57). Generally, *P. pentosaceus* undergoes the Wzx/Wzy-dependent biosynthetic pathway to produce heteropolysaccharides (58, 59). Galactose is converted to glucose-1-phosphate and subsequently to UDP-glucose, which serves as a central metabolic intermediate and gives rise to nucleotide sugars through isomerase-mediated interconversions (GDP-mannose) and the rml pathway (dTDP-rhamnose). Additionally, galactose is associated with weaker carbon catabolite repression than glucose in LAB strains, resulting in enhanced expression of genes involved in EPS biosynthesis (60).

Nitrogen is the second most essential nutrient for microbial growth and metabolism. Ammonium sulfate produces ammonium ions, which microorganisms may promptly use for cell biosynthesis, resulting in quicker growth rates and higher biomass output than alternative nitrogen sources, such as complex organic molecules (23). Yeast extract is a rich supply of easily accessible nitrogen molecules, including amino acids and peptides, which are necessary building blocks for bacteria and other microbes to produce EPS (31). Our findings supported the notion that adding yeast extract to the production medium boosted EPS levels in *L. rhamnosus* cells (61). Despite being a significant factor affecting EPS production, K_2_HPO_4_ exerted a negative effect. The observed negative effect may have resulted from the repressive effect of phosphate levels on EPS production. This was in correlation with the repressive effect of increasing phosphate levels on EPS and biofilm production in *Bacillus* strains. Elevated phosphate availability may reduce the EPS biosynthesis by redirecting the cellular resources toward growth rather than extracellular polymer production (62, 63). To minimize the negative effect of K_2_HPO_4_ level on EPS production, the K_2_HPO_4_ concentration was maintained at a lower level (0.02 g/L) for RSM experiments.

CCD-based RSM optimization of EPS synthesis employing the important independent variables galactose, ammonium sulfate, and yeast extract resulted in a 5.5-fold improvement in EPS production. Although the RSM model was statistically significant and well-fitted, the response surface plots did not display a typical closed, dome-shaped optimum. Instead, the contours showed an open, ridge-like pattern, indicating that EPS production was primarily influenced by the strong linear effect of galactose concentration. The relatively parallel contour lines further suggest that interaction effects between carbon and nitrogen sources were minor compared to the dominant role of the sugar. Although extending the galactose range might have produced a localized maximum, the experimental limits were deliberately set to prevent excessive broth viscosity and processing difficulties during subsequent formulation (64). Furthermore, investigations that found galactose had a stronger impact on EPS generation in *S. griseorubens* GD5 and *Paenibacillus polymyxa* SQR-21 documented yields of 9.50 g/L and 3.44 g/L, respectively (54, 55). The impact of ammonium sulfate at a comparable concentration (2 g/L) on the EPS synthesis of *L. plantarum* NTMI05 and NTMI20 (65). In contrast, sucrose and glucose have also been reported as optimum sugar sources for EPS production by *Leuconostoc lactis* L2, *P. pentosaceus* SL4, and *P. pentosaceus* SSC-12 (26, 51, 66).

EPS of probiotic origin serves as a prebiotic substrate and stimulant to enhance the multiplication of intestinal bacteria (67). In a recent research, different concentrations of *W. cibaria* EPS (0.1–2%) were supplemented in MRS broth to study the morphological changes, growth pattern, and antibacterial potential of *L. rhamnosus* under EPS supplemented growth conditions. EPS supplementation at a concentration of 2% in MRS agar increased viable cell count to 0.7 × 10^5^ CFU/mL compared to the *L. rhamnosus* grown in MRS agar without EPS (68). In the present study, mannose-rich EPS obtained from *P. pentosaceus* 4412 improved the growth of the probiotic consortium and sustained their survival during storage. Based on RSM experiments, the optimal conditions for enhancing the probiotic value of banana pseudostem juice with EPS as a substrate resulted in 58.0 × 10^7^ CFU/mL, with a fold increase of 3.67, compared with the unoptimized fermentation, which yielded 15.8 × 10^7^ CFU/mL. The color of the FBPJ was observed as pale creamy-whitish color.

The lower probiotic viable count of 1.02 × 10^7^ CFU/mL in the no-EPS control highlights the role of EPS in promoting the growth of probiotic bacteria and fermentation of BPJ. In the present study, the term “prebiotic potential” refers to the capacity of EPS-4412, isolated from the probiotic producer strain, to serve as a fermentable, non-digestible substrate that selectively enhances the growth and viability of probiotic microorganisms during banana pseudostem juice fermentation. The RSM results demonstrate that EPS-4412 was most effective at lower concentrations (0.1%), evidencing the modulatory growth enhancer and prebiotic stimulant potential rather than a bulk carbohydrate source. The relatively low molecular weight of EPS-4412 may also contribute to its fermentability than that of more complex, higher molecular weight polysaccharides. A nonlinear trend was observed in EPS concentration and viable cell count, where the highest and lowest EPS concentrations were associated with higher viable cell counts, whereas the medium EPS concentration was not. This nonlinear trend in viable cell count is influenced by interactions among EPS concentration, inoculum size, and incubation time, rather than by EPS concentration alone. At higher inoculum levels and shorter incubation times, higher viable counts were observed in the presence of both higher and lower concentrations of EPS. Whereas in the midrange of EPS, the combination of mid inoculum size and incubation time consistently resulted in a lower viable cell count, likely reflecting early stationary-phase dynamics and metabolic equilibrium. These findings suggest that probiotic growth in the fermentation system is governed by density-dependent EPS interactions rather than by a simple linear concentration-response relationship.

EPS generated by *Leuconostoc mesenteroides* F27, a strain isolated from fermented milk, was employed as a biological supplement to elevate the functional quality of yogurt. At a concentration of 250 mg/L, *L. mesenteroides* F27 EPS stimulated probiotic LAB growth, including *S. thermophilus*, *L. bulgaricus*, *L. plantarum* C182, and *L. sakei* CJLS03 in yogurt with excellent rheological and antioxidant potential (69). Fermentation of pureed carrot with EPS-producing LAB improved texture and flexibility while also creating a pleasant odor and flavor. This indicated that EPS-producing LAB-based fermentation of vegetable products might be a substantial alternative to hydrocolloid additions as texturizers (70). Samples with low colony counts retained their viability, achieving the criterion for probiotic viability at levels of 7–8 log CFU/mL, which is closer to the aim of 9 log CFU/mL (38, 71). Based on the colony counts from the 20 optimized runs, the samples were further analyzed. The molecular weight, monosaccharide composition, glycosidic linkage, and functional groups of EPSs determine their utilization by a probiotic strain (72).

The molecular structure of EPS-4412, which is rich in mannose, glucose, and rhamnose, serves as an excellent substrate for both probiotic proliferation and beneficial metabolite synthesis in symbiotic systems (19, 73). The glycosidic linkages such as *α*-(1,2), α-(1,3), *β*-(1,4), and β-(1,2) contribute to the complex structure of EPS-4412 which impedes their break down by LAB strains while still stimulates their growth and promotes fermentation due to its low concentration as well as molecular weight (19, 72, 73). The α-(1,3) and α-(1,6) glycosidic linkages of glucan type EPS obtained from *L. brevis* ED25 act as a prebiotic growth stimulant of *Lactobacillus* GG in the chocolate pudding model (74). This metabolic conversion demonstrates the functional synergy between EPS-4412 supplementation and increased probiotic viability through the formation of health-promoting SCFAs (19). As a result, the probiotic consortium metabolized EPS as a fermentable substrate to elevate growth, rather than exhibiting a synergistic effect.

Generally, the pH of banana pseudostem juice varies from 4.22 to 7.8 (75). The pH of FBPJ17 (3.63 ± 0.25) was similar to the pH of water kefir prepared from pomace and juice (3.39 ± 0.01 and 3.59 ± 0.01), respectively. The variation in acid production across LAB strains affects the pH value of fermented fruit and vegetable juices (76). Acidic pH promotes the formation of organic acids and the breakdown of carbohydrates by probiotic bacteria such as *Lactobacillus, Leuconostoc, Pediococcus, Lactococcus*, and *Enterococcus* (71, 77). These LAB and EPSs facilitated the eradication of spoilage indicators and infections by decreasing the pH of the FBPJ (28). As pH increases, titratable acidity decreases, comparable to the results of fermented coconut milk (27). The mixed cultures metabolized the polysaccharide and other sugars in the sample as growth supplements, as demonstrated by lower values compared to FBPJ15 (62.87 ± 1.91%) and FBPJ5 (47.03 ± 1.57%). These findings were consistent with the sugar consumption of *Lactobacillus* strains that fermented the jujube-wolfberry composite juice (37). Polyphenols are key bioactive compounds in plant-based functional foods, enhancing flavor and offering substantial health benefits (37). Interestingly, the utilization of EPS by microbial consortium has enhanced the production of phenolic compounds, evidenced by increased TPC values. The phenolic content of fermented kiwifruit juice was elevated to 60 mg GAE/L, which is consistent and greater than our present study (38).

VOCs are essential for assessing the organoleptic quality of fermented foods to ensure customer acceptance (76). This mixed LAB fermentation contributes to the advancement of the biological properties and flavor-inducing volatile compound production (71). This mixed LAB fermentation contributes to the advancement of the biological properties and flavor-inducing volatile compounds production (11). LAB-mediated fermentation produces acids, aldehydes, alcohols, esters, ketones, and other compounds that are associated with the fragrance and sensory aspects of vegetables and fruit juice (38). Microbial consortia, EPS, BPS juice, and fermentation duration are the key elements that contribute to the generation of VOCs. The fermentation of carbohydrates by LAB produces acid, which increases the shelf life and safety of functional foods (78). Interestingly, fragrance molecules improve the taste, flavor, and texture of plant-based dairy substitutes that are comparable to milk fermented products (57). The primary flavor chemicals classified as acids, alcohols, ketones, and esters are as follows: 2,5-dimethyl-4-hydroxy-3(2H)-furanone, dodecanoic acid, ethenyl ester, hexadecanoic acid, 2-oxo-, and methyl ester provide fruity and floral smells. 1-(acetyloxy)-2-butanone, O-decyl-hydroxylamine, 1,3(z),13-tetradecatriene, (z)-4-tridecene, 1-tridecene, and dichloroacetic acid, nonyl ester.

The influence of LAB fermentation is demonstrated by the formation of physiologically active compounds, which play an important role in therapeutic aspects (39). In FBPJ15, volatile flavor molecules such as oxalic acid, heptyl propyl ester, 3-buten-2-ol, 1-(acetyloxy)-2-butanone, 2,5-dimethyl-4-hydroxy-3(2 h)-furanone, and 6-methyl-undecane are regarded as major fragrance contributors. However, these acids, esters, alcohols, ketones, and hydrocarbons are in lower concentrations than the other three samples. A microbial consortium consisting of *Lpb. pentosus* AKVIT2, *Lpb. plantarum* BFW MKVIT02, and *P. pentosaceus* 4412 produce flavor in fermented food matrices (78). This probiotic consortium utilized mannose and glucose present in EPS-4412 as a carbon source during fermentation and converted it into short-chain fatty acids (SCFAs) such as butyric acid, acetic acid, and propionic acid derivatives, which promoted the consortium’s development (73, 79). In a similar study, the mannose, glucose, galactose, and galacturonic acid belonging to EPS were extracted from *Lpb. plantarum* YT013 was fermented to produce SCFAs including N-butyric acid, acetic and propionic acid (73).

Alcohols and esters such as 2-decen-1-ol, (e)-2-nonen-1-ol, 2-butyl-1-octanol, oxalic acid, dodecyl hexyl ester, and oxalic acid, allyl pentadecyl ester produced in FBPJ17 and FBPJ5 samples contribute to floral, fruity, and sweet aroma (33, 80). Hexadecanal, undecanal, and octadecanal present in FBPJ5 serve as fragrance and flavor ingredients, which have highly to the improvement of organoleptic properties. These results are in accordance with the similar compounds generated during *P. acidilactici* BD 16 (alaD^+^) stimulated fermentation of buttermilk and soymilk (39). 1-(ethenyloxy)-octadecane and 4,4-dimethyl-2-pentanone (ketones) have promoted flavor, whereas dotriacontane, oxalic acid, allyl pentadecyl ester, and DL-3-aminobutyric acid are found in FBPJ17 and FBPJ5, which have antimicrobial, termiticidal, and maggoticidal antispasmodic properties (81). In the FBPJ17 sample, 2-hexyl-1-decanol and 11-tridecen-1-ol possess antioxidant properties (39), whereas E-2-tetradecen-1-ol encompasses anti-inflammatory, antihepatotoxicity, and anti-cancer potential (82). In an earlier study, GC–MS analysis of fermented millet substrate using *P. pentosaceus* RZ01 and *Lacticaseibacillus paracasei* RZ02 revealed the occurrence of oleic acid, 3-(octadecyloxy) propyl ester, 7-methyl-Z-tetradecen-1-ol acetate, and n-hexadecanoic acid, closely related to our current results (82). N-[4-bromo-n-butyl]-2-piperidinone is an alkaloid or *δ*-valerolactam found in FBPJ18, FBPJ17, and FBPJ5, respectively. This secondary metabolite exhibits remarkable antibacterial, anti-inflammatory, and antioxidant activities (83). The presence of lactones such as trans and cis-hexahydro(5H)-cyclohepta-1,4-dioxin-2(3H)-one reflects the lipid oxidation of fatty acids and generates a rich fruity flavor (78).

The agar well diffusion technique analyzed the compatibility of FBPJ toward the probiotic strains. As there was no zone of inhibition around the wells added with EPS based FBPJ, a positive prebiotic-probiotic compatibility can be observed. Also, FBPJ can be considered biocompatible in maintaining the viability of the probiotic strains (67). The LAB strains are capable of secreting various bioactive substances like polysaccharides, peptides, organic acids, vitamins, and bacteriocins during the fermentation period. Based on the literature, EPS plays an important role in counteracting various pathogens. Additionally, antimicrobial compounds like [(Aminocarbonyl)amino] oxo-acetic acid, 2-oxo-propanoic acid, and DL-3-aminobutyric acid were responsible for inhibiting the growth of *S. enterica* MTCC 1164 (76). The FBPJ has exhibited antagonistic activity only against this enteric pathogen, and its ability to encounter the other food-borne infection-causing pathogens should be evaluated. In a recent study, peach juice fermented using *L. acidophilus* PTCC 1643 and *L. fermentum* PTCC 1744 showed antibacterial activity against a food-borne pathogen, *S. flexneri* PTCC 1865, confirming its health-promoting potential (84).

The fermentation with three LAB using EPS as substrate has remarkably promoted the antioxidant activity of banana pseudostem juice. The DPPH radical scavenging activity of jujube–wolfberry composite juice fermented using *L. plantarum* 9–2 (LP9-2) was increased to 92.85 ± 0.05% than our findings (74.43 ± 0.96%) after 72 h of fermentation, compared to non-fermented juice (37). The DPPH radical scavenging activity of fermented goji juice with multiple probiotic strains is comparable to the radical scavenging potential of the FBPJ17 sample (32). The bacterial consortium supplemented with EPSs has significantly contributed to the DPPH radical scavenging activity (76). Peach juice fermented with *Lactobacillus* strains exhibited a reducing power (from 1.5 to 2), compared to FBPJ17 (0.72 ± 0.058) after 48 h of fermentation, corresponding to its phenolic content (84). The phenolic content and reducing power capacity are interrelated. Our results followed the same pattern of superoxide radical scavenging activity as that observed in fermented peach juice produced by *Lactobacillus* strains. LAB-mediated fermentation generates reductones, which interact with free radicals to neutralize their action (84). The DPPH activity and reducing power are primarily attributed to the bound and free forms of phenolic compounds (32). The superoxide radical scavenging activity of fermented peach juice employing *L. acidophilus* and *L. fermentum* reached 74.27 and 67.50% after 48 h of fermentation. Our results are slightly lower than the aforementioned outcomes, where FBPJ17 has achieved 65.6 ± 1.1% superoxide scavenging potential (84).

Fermented foods (FBPJ) with *L. plantarum* BFW MKVIT02, *L. pentosus* AKVIT2, and *P. pentosaceus* 4412 can augment antioxidant levels by producing *β*-glucosidase, which converts phenolic hydrolases into aglycones (85). The organic acids, alcohols, and esters found in the VOC study, such as 2-oxo-propanoic acid, 1-tridecen-1-ol, 2-hexyl-1-decanol, octadecanoic acid, and 2-oxo-hexadecanoic acid, methyl ester, were linked to FBPJ17’s high antioxidant properties. However, investigation on *in vivo* models and a mechanistic evaluation of the biological potential of FPBJ should be conducted in future studies to correlate its importance for human consumption. BPS juice was converted into a functional beverage during fermentation, and the addition of EPS allowed for the evaluation of its prebiotic stimulant potential to improve LAB consortium growth, viability, and synergistic activity, all of which are critical components in developing next-generation symbiotic and value-added functional foods. This integrated strategy promotes the value of an underused agricultural resource while also advancing research into the health advantages of EPS in probiotic systems. FBPJ may be a source of potential prebiotics, probiotics, and antioxidants, with a pleasing mouthfeel for lactose-intolerant customers (76).

The present study begins with the statistical optimization of EPS production by *P. pentosaceus* 4412 and its application as a functional prebiotic component and prebiotic stimulant in the fermentation of banana pseudostem juice. The integration of statistical techniques with the FBPJ aided in optimizing the conditions necessary for optimum prebiotic activity of EPS-4412. Further, the physicochemical characterization, GC–MS profiling for VOC analysis, and bioactivity assessment represent a major strength of the present research. To the best of our knowledge, this is the first study to integrate statistical optimization of EPS production with functional validation as a probiotic growth stimulant in the fermentation of banana pseudostem juice. The novelty of the current study lies in the incorporation of EPS optimization, fermentation science, and valorization of agro byproduct (banana pseudostem).

A few limitations remain to be addressed for further confirmation of the present results. The prebiotic potential and the biological activities of FBPJ were evaluated using *in vitro* assays. Although these methods provide the preliminary evidence of functional potential, their variability in the human gastrointestinal environment emphasizes the need for further *in vivo* studies or simulated gut models to confirm the relevance of the findings. As most typically marketed probiotic beverages are stored at chilled conditions, the present research assessed storage stability at 4 °C. To improve marketability and consumer feasibility, future studies could evaluate the product’s stability at ambient temperatures to minimize the need for cold-chain infrastructure. Even though 0.1% EPS supported a significant probiotic viability and notable bioactivity, the underlying mechanism was not investigated in this study and remains an important direction for future research.

## 5 Conclusion

Initially, the enhancement of EPS production was achieved by sequentially applying OFAT, PB, and RSM approaches, which identified the most significant variables, including galactose (36.81 g/L), yeast extract (5 g/L), and ammonium sulfate (2 g/L) that influenced EPS production. The highest production of EPS-4412 was 3.46 ± 0.16 g/L, indicating a 5.5-fold increase relative to unoptimized conditions. Fermentation of BPJ using CCD-based RSM to optimize three parameters: the concentration of EPS-4412, the inoculum size of the consortium, and the fermentation time. The viable colony counts, pH, titratable acidity, sugar content, and total phenolic content (TPC) were determined for the FBPJ samples. Based on the GC–MS analysis, esters, alcohols, and ketones have highly promoted the flavor and aroma of the optimized FBPJ17 compared to other fermented and unfermented BPS juice. The antibacterial and antioxidant activities of FBPJ17 were higher than those of the other samples. Thus, the fermentation of banana pseudostem juice with the integration of a LAB consortium (probiotic) and EPS-4412 (prebiotic stimulant) represents a significant symbiotic, value-added, and nutrition-rich beverage.

## Data Availability

The original contributions presented in the study are publicly available. The data on strain identification can be found in the NCBI GenBank repository under the accession numbers OR095723 (*Pediococcus pentosaceus* 4412), OR088203 (*Lactobacillus plantarum* BFW MKVIT02), and PV603401 (*Lactiplantibacillus pentosus* AKVIT2).

## References

[1] Angelin J Kavitha M. Exopolysaccharides from probiotic bacteria and their health potential. Int J Biol Macromol. (2020) 162:853–65. doi: 10.1016/j.ijbiomac.2020.06.190, 32585269 PMC7308007

[2] Nithya A Misra S Panigrahi C Dalbhagat CG Mishra HN. Probiotic potential of fermented foods and their role in non-communicable diseases management: an understanding through recent clinical evidences. Food Chem Advan. (2023) 3:100381. doi: 10.1016/j.focha.2023.100381

[3] Lee M. Potential probiotic properties of exopolysaccharide-producing *Lacticaseibacillus paracasei* EPS DA-BACS and prebiotic activity of its exopolysaccharide. Microorganisms. (2022) 10:1–20. doi: 10.3390/microorganisms10122431 PMC9787920 36557684

[4] Ju J Ju J-H Jeon S-G Heo S-Y Kim J-S Jo M-H . Synbiotics production using *Lactobacillus reuteri* EC01, a strain that produces alternan-type exopolysaccharide. LWT. (2023) 182:114814. doi: 10.1016/j.lwt.2023.114814

[5] Erginkaya Z Konuray-Altun G. Potential biotherapeutic properties of lactic acid bacteria in foods. Food Biosci. (2022) 46:101544. doi: 10.1016/j.fbio.2022.101544

[6] Wang B Song Q Zhao F Han Y Zhou Z. Production optimization, partial characterization and properties of an exopolysaccharide from *Lactobacillus sakei* L3. Int J Biol Macromol. (2019) 141:21–28. doi: 10.1016/j.ijbiomac.2019.08.241, 31473313

[7] Prete R Alam MK Perpetuini G Perla C Pittia P Corsetti A. Lactic acid bacteria exopolysaccharides producers: a sustainable tool for functional foods. Foods. (2021) 10:1–27. doi: 10.3390/foods10071653, 34359523 PMC8305620

[8] Singh V Haque S Niwas R Srivastava A Pasupuleti M Tripathi CKM. Strategies for fermentation medium optimization: an in-depth review. Front Microbiol. (2017) 7. doi: 10.3389/fmicb.2016.02087, 28111566 PMC5216682

[9] Victoria O Folakemi A Olugbemiga S. Unveiling the role of functional foods with emphasis on prebiotics and probiotics in human health: a review. J Funct Foods. (2024) 119:106337. doi: 10.1016/j.jff.2024.106337

[10] Liu W Pu X Sun J Shi X Cheng W Wang B. Effect of *Lactobacillus plantarum* on functional characteristics and flavor profile of fermented walnut milk. LWT. (2022) 160:113254. doi: 10.1016/j.lwt.2022.113254

[11] Yi C Li Y Zhu H Liu Y Quan K. Effect of *Lactobacillus plantarum* fermentation on the volatile flavors of mung beans. LWT. (2021) 146:111434. doi: 10.1016/j.lwt.2021.111434

[12] Pan X Zhang S Xu X Lao F Wu J. Volatile and non-volatile profiles in jujube pulp co-fermented with lactic acid bacteria. LWT. (2022) 154:112772. doi: 10.1016/j.lwt.2021.112772

[13] Chakraborty R Sabruna S Roy R Majumdar S Roy S. Banana pseudostem substitution in wheat flour biscuits enriches the nutritional and antioxidative properties with considerable acceptability. SN Appl Sci. (2021) 3:1–12. doi: 10.1007/s42452-020-03988-1

[14] Pillai GS Morya S Khalid W Khalid MZ Almalki RS Siddeeg A. Banana pseudostem: an undiscovered fiber enriched sustainable functional food. J Nat Fibers. (2024) 21:1–15. doi: 10.1080/15440478.2024.2304004

[15] Shetty N. Development of fermented beverages from banana pseudo stem core juice enriched with honey and whey and their shelf-life assessment. Pharma Innovation J. (2022) 11:355–61.

[16] Ripari V. Techno-functional role of exopolysaccharides in cereal-based, yogurt-like beverages. Beverages. (2019) 5:16. doi: 10.3390/beverages5010016

[17] Song Y Song YR Lee CM Lee SH Baik SH. Evaluation of probiotic properties of *Pediococcus acidilactici* M76 producing functional exopolysaccharides and its lactic acid fermentation of black raspberry extract. Microorganisms. (2021) 9:1364. doi: 10.3390/microorganisms9071364, 34201704 PMC8304599

[18] Lahiri D Nag M Mukherjee D Garai S Banerjee R Ray RR. Recent trends in approaches for optimization of process parameters for the production of microbial cellulase from wastes. Environ Sustain. (2021) 4:273–84. doi: 10.1007/s42398-021-00189-3

[19] Angelin J Kavitha M. Structural characterization and in vitro anti-inflammatory activity of exopolysaccharide produced by Pediococcus pentosaceus 4412. Int Immunopharmacol. (2025) 150:114301. doi: 10.1016/j.intimp.2025.114301, 39970712

[20] Wang J Zhang J Guo H Cheng Q Abbas Z Tong Y . Optimization of exopolysaccharide produced by *Lactobacillus plantarum* R301 and its antioxidant and anti-inflammatory activities. Foods. (2023) 12:2481. doi: 10.3390/foods12132481, 37444218 PMC10340397

[21] Dubois M Gilles KA Hamilton JK Rebers PA Smith F. Colorimetric method for determination of sugars and related substances. Anal Chem. (1956) 28:350–6.

[22] Chen L Gu Q Zhou T. Statistical optimization of novel medium to maximize the yield of exopolysaccharide from Lacticaseibacillus rhamnosus ZFM216 and its immunomodulatory activity. Front Nutr. (2022) 9:924495. doi: 10.3389/fnut.2022.924495, 35719166 PMC9201479

[23] Arayes MA Mabrouk MEM Sabry SA Abdella B. Exopolysaccharide production from Alkalibacillus sp. w3: statistical optimization and biological activity. Biologia. (2023) 78:229–40. doi: 10.1007/s11756-022-01233-1

[24] Iswareya Lakshimi V Kavitha M. Response surface optimization of solvent-tolerant cold-active lipase production by *Pseudomonas* sp. VITCLP4. Catal Lett. (2025) 155:21. doi: 10.1007/s10562-024-04848-y

[25] Elmansy EA Elkady EM Asker MS Abdallah NA Khalil BE Amer S. Improved production of lactiplantibacillus plantarum RO30 exopolysaccharide (REPS) by optimization of process parameters through statistical experimental designs. BMC Microbiol. (2023) 23:361. doi: 10.1186/s12866-023-03117-z, 37993835 PMC10664612

[26] Tian J Fan Y Li X Cui K Zhang J. Optimising the production of exopolysaccharides from *Pediococcus pentosaceus* SSC-12 and characterizing its properties. LWT. (2025) 226:117967. doi: 10.1016/j.lwt.2025.117967

[27] Qadi WSM Mediani A Benchoula K Wong EH Misnan NM Sani NA. Characterization of physicochemical, biological, and chemical changes associated with coconut milk fermentation and correlation revealed by 1H NMR-based metabolomics. Foods. (2023) 12:1–24. doi: 10.3390/foods12101971, 37238789 PMC10217123

[28] Yang J Sun Y Gao T Wu Y Sun H Zhu Q . Fermentation and storage characteristics of “Fuji” apple juice using *Lactobacillus acidophilus*, *Lactobacillus casei* and *Lactobacillus plantarum*: microbial growth, metabolism of bioactives and in vitro bioactivities. Front Nutr. (2022) 9:1–14. doi: 10.3389/fnut.2022.833906, 35223961 PMC8864132

[29] Elmansy EA Elkady EM Asker MS Abdou AM Abdallah NA Amer SK. Exopolysaccharide produced by *Lactiplantibacillus plantarum* RO30 isolated from Romi cheese: characterization, antioxidant and burn healing activity. World J Microbiol Biotechnol. (2022) 38:245–18. doi: 10.1007/s11274-022-03439-6, 36287274 PMC9605930

[30] Rastogi S Mittal V Singh A. In vitro evaluation of probiotic potential and safety assessment of *Lactobacillus mucosae* strains isolated from donkey’s lactation. Probiotics Antimicrob Proteins. (2019) 12:1045–56. doi: 10.1007/s12602-019-09610-0, 31713771

[31] Zhang L Zhao B En CL. Optimization of biosynthesis conditions for the production of exopolysaccharides by *Lactobacillus plantarum* SP8 and the exopolysaccharides antioxidant activity test. Indian J Microbiol. (2020) 60:334–45. doi: 10.1007/s12088-020-00865-8, 32647393 PMC7329955

[32] Liu Y Cheng H Liu H Ma R Ma J Fang H. Fermentation by multiple bacterial strains improves the production of bioactive compounds and antioxidant activity of goji juice. Molecules. (2019) 24:1–14. doi: 10.3390/molecules24193519, 31569407 PMC6804111

[33] Aminianfar A Hossein M Azimi F. Comprehensive characterization of volatile compounds in Iranian black teas using chemometric analysis of GC-MS fingerprints. Food Chem X. (2024) 24:101859. doi: 10.1016/j.fochx.2024.101859, 39403300 PMC11471522

[34] Lin F Cai F Luo B Gu R Ahmed S. Variation of microbiological and biochemical profiles of Laowo dry-cured ham, an indigenous fermented food, during ripening by GC-TOF-MS and UPLC-QTOF-MS. J Agric Food Chem. (2020) 68:8925–35. doi: 10.1021/acs.jafc.0c03254, 32706588

[35] Chen G Chen G-L Lin B Zheng F-J Fang X-C Yang Y-X . Assessment of acid production efficiency and aroma volatile compounds by immobilized fermentation of different carriers on sugarcane original vinegar. LWT. (2024) 203:116285. doi: 10.1016/j.lwt.2024.116285

[36] Huang Y Huang Y-y Yu J-j Zhou Q-y Sun L-n Liu D-m . Preparation of yogurt-flavored bases by mixed lactic acid bacteria with the addition of lipase. LWT. (2020) 131:109577. doi: 10.1016/j.lwt.2020.109577

[37] Zhao X Tang F Cai W Peng B Zhang P Shan C. Effect of fermentation by lactic acid bacteria on the phenolic composition, antioxidant activity, and flavor substances of jujube – wolfberry composite juice. LWT. (2023) 184:114884. doi: 10.1016/j.lwt.2023.114884

[38] Lan T Lv X Zhao Q Lei Y Gao C Yuan Q . Optimization of strains for fermentation of kiwifruit juice and effects of mono- and mixed culture fermentation on its sensory and aroma profiles. Food Chem X. (2023) 17:100595. doi: 10.1016/j.fochx.2023.100595, 36824148 PMC9941363

[39] Sharma A Noda M Sugiyama M Ahmad A Kaur B. Production of functional buttermilk and soymilk using *Pediococcus acidilactici* BD16 (alaD +). Molecules. (2021) 26:1–25. doi: 10.3390/molecules26154671 PMC8347781 34361824

[40] Zhang K Zhang TT Guo RR Ye Q Zhao HL Huang XH. The regulation of key flavor of traditional fermented food by microbial metabolism: a review. Food Chem X. (2023) 19:100871. doi: 10.1016/j.fochx.2023.100871, 37780239 PMC10534219

[41] Yuan X Jiang W Zhang D. Textural, sensory and volatile compounds analyses in formulations of sausages analogue elaborated with edible. Foods. (2022) 11:1–14. doi: 10.3390/foods11010052 PMC8750815 35010178

[42] Rajendran S Silcock P Bremer P. Flavour volatiles of fermented vegetable and fruit substrates: a review. Molecules. (2023) 28:1–36. doi: 10.3390/molecules28073236, 37049998 PMC10096934

[43] Adeyinka J. Metabolite pro fi le of Bambara groundnut (*Vigna subterranea*) and dawadawa (an African fermented condiment) investigation using gas chromatography high resolution time-of- fl ight mass spectrometry (GC-HRTOF-MS). HLY. (2021) 7:e06666. doi: 10.1016/j.heliyon.2021.e06666, 33889778 PMC8050003

[44] Guo Q Peng J Zhao J Yue J Huang Y Shao B. Correlation between microbial communities and volatile flavor compounds in the fermentation of semen Sojae Praeparatum. LWT. (2024) 198:116009. doi: 10.1016/j.lwt.2024.116009

[45] Melini F Melini V. Role of microbial fermentation in the bio-production of food aroma compounds from vegetable waste. Fermentation. (2024) 10:1–23. doi: 10.3390/fermentation10030132

[46] Sabri NM. Microbial populations, sensory, and volatile compounds profiling of local cooked rice. Food Qual Saf. (2024) 8:1–12. doi: 10.1093/fqsafe/fyad065

[47] Bhowmik B Afrin S Jui AH Bhuiyan RH Rashid MM Miah MAS . Exploring the purification, characterization, and industrial applications of exopolysaccharide (EPS) from *Bacillus amyloliquefaciens* strain BDIFST240014. Mol Biol Rep. (2025) 52:51–15. doi: 10.1007/s11033-024-10173-9, 39680227

[48] Islam MF Islam S Miah MAS Bhuiyan MNI Abedin N Mondol MMH . Quality assessment and sensory evaluation of green banana starch enriched instant noodles. Appl Food Res. (2024) 4:100431. doi: 10.1016/j.afres.2024.100431

[49] Islam S Islam MF Bhuiyan MNI Tisa KJ Kibria A Bhuiyan MHR . Integrating green banana and cauliflower into whole wheat pasta: approaches to enhance nutritional benefits while maintaining quality. Food Chem Advan. (2025) 6:100933. doi: 10.1016/j.focha.2025.100933

[50] Ahmed Z Mehmood T Ferheen I Noori AW Almansouri M Waseem M. Optimization of exopolysaccharide produced by *L. kefiranofaciens* ZW3 using response surface methodology. Int J Food Prop. (2023) 26:2285–93. doi: 10.1080/10942912.2023.2245577

[51] Jiang J Guo S Ping W Zhao D Ge J. Optimization production of exopolysaccharide from *Leuconostoc lactis* L2 and its partial characterization. Int J Biol Macromol. (2020) 159:630–9. doi: 10.1016/j.ijbiomac.2020.05.101, 32439434

[52] Tilwani YM Lakra AK Domdi L Yadav S Jha N Arul V. Optimization and physicochemical characterization of low molecular Levan from *Enterococcus faecium* MC-5 having potential biological activities. Process Biochem. (2021) 110:282–91. doi: 10.1016/j.procbio.2021.08.021

[53] Zhao D Liu L Jiang J Guo S Ping W Ge J. The response surface optimization of exopolysaccharide produced by *Weissella confusa* XG-3 and its rheological property. Prep Biochem Biotechnol. (2020) 50:1014–22. doi: 10.1080/10826068.2020.1780609, 32589090

[54] Vinothini G Latha S Arulmozhi M Dhanasekaran D. Statistical optimization, physio-chemical and bio-functional attributes of a novel exopolysaccharide from probiotic *Streptomyces griseorubens* GD5. Int J Biol Macromol. (2019) 134:575–87. doi: 10.1016/j.ijbiomac.2019.05.011, 31067487

[55] Raza W Makeen K Wang Y Xu Y Qirong S. Optimization, purification, characterization and antioxidant activity of an extracellular polysaccharide produced by *Paenibacillus polymyxa* SQR-21. Bioresour Technol. (2011) 102:6095–103. doi: 10.1016/j.biortech.2011.02.033, 21392978

[56] Peter SB Qiao Z Godspower HN Ajeje SB Xu M Zhang X . Biotechnological innovations and therapeutic application of *Pediococcus* and lactic acid bacteria: the next-generation microorganism. Front Bioeng Biotechnol. (2022) 9:1–13. doi: 10.3389/fbioe.2021.802031, 35237589 PMC8883390

[57] Xiao H Sedó Molina GE Tovar M Minh Quoc H Hansen EB Bang-Berthelsen CH. Isolation and characterization of plant-based lactic acid bacteria from spontaneously fermented foods using a new modified medium. LWT. (2024) 192:1–11. doi: 10.1016/j.lwt.2023.115695

[58] Halfawy NME Zaghloul EH. Functional and genomic evaluation of novel exopolysaccharide produced by marine *Pediococcus pentosaceus* E3 with antidiabetic, anticancer, and anti-inflammatory potentials. BMC Microbiol. (2025) 25:628. doi: 10.1186/s12866-025-04370-0, 41044694 PMC12492903

[59] Zhang Y Kaushik N Park P Liu R Liu Y. *Pediococcus pentosaceus* in fermented foods: probiotic properties and a multi-omics framework for applications. Trends Food Sci Technol. (2025) 163:105187. doi: 10.1016/j.tifs.2025.105187

[60] Fuso A Bancalari E Castellone V Caligiani A Gatti M Bottari B. Feeding lactic acid bacteria with different sugars: effect on. Foods. (2023) 12:15–6. doi: 10.3390/foods12010215 PMC9819028 36613431

[61] Oleksy-sobczak M. Optimization of media composition to maximize the yield of exopolysaccharides production by *Lactobacillus rhamnosus* strains. Probiotics Antimicrobial Proteins. (2020) 12:774–83. doi: 10.1007/s12602-019-09581-2, 31410767 PMC7306023

[62] Çam S Badıllı İ. The effect of NaCl, pH, and phosphate on biofilm formation and exopolysaccharide production by high biofilm producers of Bacillus strains. Folia Microbiol. (2024) 69:613–24. doi: 10.1007/s12223-023-01101-8, 37897595

[63] Li Y Cao X Chai Y Chen R Zhao Y Borriss R . A phosphate starvation induced small RNA promotes Bacillus biofilm formation. NPJ Biofilms Microbiomes. (2024) 10:115–2. doi: 10.1038/s41522-024-00586-6, 39472585 PMC11522486

[64] Freitas F Alves VD Reis MAM. Advances in bacterial exopolysaccharides: from production to biotechnological applications. Trends Biotechnol. (2011) 29:388–98. doi: 10.1016/j.tibtech.2011.03.008, 21561675

[65] Imran MYM Reehana N Jayaraj KA Ahamed AAP Dhanasekaran D Thajuddin N . Statistical optimization of exopolysaccharide production by *Lactobacillus plantarum* NTMI05 and NTMI20. Int J Biol Macromol. (2016) 93:731–45. doi: 10.1016/j.ijbiomac.2016.09.007, 27601132

[66] Puspitasari D.A. (2025). ‘Optimization on production and technological properties of exopolysaccharide from *Pediococcus pentosaceus* SL4 isolated from sourdough Tomi- tomi (*Flacourtia Inermis* Roxb)’, pp. 1–27. doi: 10.21203/rs.3.rs-7555681/v1 41876807

[67] Bouzaiene T Mohamedhen Vall M Ziadi M Ben Rejeb I Yangui I Aydi A . Exopolysaccharides from *Lactiplantibacillus plantarum* C7 exhibited antibacterial, antioxidant, anti-enzymatic, and prebiotic activities. Fermentation. (2024) 10:339. doi: 10.3390/fermentation10070339

[68] Park S Saravanakumar K Sathiyaseelan A Han KS Lee J Wang MH. Polysaccharides of *Weissella cibaria* act as a prebiotic to enhance the probiotic potential of *Lactobacillus rhamnosus*. Appl Biochem Biotechnol. (2023) 195:3928–40. doi: 10.1007/s12010-022-04104-2, 35947292

[69] Nemati V Mozafarpour R. Exopolysaccharides isolated from fermented milk-associated lactic acid bacteria and applied to produce functional value-added probiotic yogurt. LWT. (2024) 199:116116. doi: 10.1016/j.lwt.2024.116116

[70] Juvonen R Honkapää K Maina NH Shi Q Viljanen K Maaheimo H . The impact of fermentation with exopolysaccharide producing lactic acid bacteria on rheological, chemical and sensory properties of pureed carrots (*Daucus carota* L.). Int J Food Microbiol. (2015) 207:109–18. doi: 10.1016/j.ijfoodmicro.2015.04.031, 26001525

[71] Wang Y Li W Wang X Hu Q Kong J Wang X . Investigation of volatile compounds during fermentation of Elaeagnus HN-3 and *Lacticaseibacillus paracasei* YL-29. Food Chem X. (2024) 21:1–14. doi: 10.1016/j.fochx.2024.101171, 38370297 PMC10869281

[72] Yu L Ye G Qi X Yang Y Zhou B Zhang Y . Purification, characterization and probiotic proliferation effect of exopolysaccharides produced by *Lactiplantibacillus plantarum* HDC-01 isolated from sauerkraut. Front Microbiol. (2023) 14:1–13. doi: 10.3389/fmicb.2023.1210302, 37440877 PMC10333699

[73] Chen M Zhang H Yang R Wang X du L Yue Y . Structural analysis and prebiotic properties of the polysaccharides produced by Lactiplantibacillus plantarum YT013. Food Chem X. (2025) 28:102600. doi: 10.1016/j.fochx.2025.102600, 40535593 PMC12173702

[74] Ispirli H Demirbas F Derti E. Glucan type exopolysaccharide (EPS) shows prebiotic effect and reduces syneresis in chocolate pudding. J Food Sci Technol. (2018) 55:3821–6. doi: 10.1007/s13197-018-3181-3, 30150842 PMC6098793

[75] Islam MS Kasim S Amin AM Hun TG Alam MK Haque MA. Banana-pseudostem sap growing media as a novel source of phytochemicals and mineral nutrients: influence on seedling growth of sweet corn. Chil J Agric Res. (2022) 82:135–45. doi: 10.4067/S0718-58392022000100135

[76] Esatbeyoglu T Fischer A Legler ADS Oner ME Wolken HF Köpsel M . Physical, chemical, and sensory properties of water kefir produced from *Aronia melanocarpa* juice and pomace. Food Chem X. (2023) 18:1–11. doi: 10.1016/j.fochx.2023.100683, 37138825 PMC10149414

[77] Elhalis H See XY Osen R. The potentials and challenges of using fermentation to improve the sensory quality of plant-based meat analogs. Front Microbiol. (2023) 14:1–21. doi: 10.3389/fmicb.2023.1267227, 37860141 PMC10582269

[78] Hu Y Zhang L Wen R Chen Q Kong B. Role of lactic acid bacteria in flavor development in traditional Chinese fermented foods: a review. Crit Rev Food Sci Nutr. (2022) 62:2741–55. doi: 10.1080/10408398.2020.1858269, 33377402

[79] Gao Z Wu C Wu J Zhu L Gao M Wang Z . Antioxidant and anti-inflammatory properties of an aminoglycan-rich exopolysaccharide from the submerged fermentation of *Bacillus thuringiensis*. Int J Biol Macromol. (2022) 220:1010–20. doi: 10.1016/j.ijbiomac.2022.08.116, 36030974

[80] Yuan X Wang T Sun L Qiao Z Pan H Zhong Y . Food chemistry: X recent advances of fermented fruits: a review on strains, fermentation strategies, and functional activities. Food Chem X. (2024) 22:101482. doi: 10.1016/j.fochx.2024.101482, 38817978 PMC11137363

[81] Padhan B Poddar K Sarkar D Sarkar A. Production, purification, and process optimization of intracellular pigment from novel psychrotolerant *Paenibacillus* sp. BPW19. Biotechnol Rep. (2021) 29:e00592. doi: 10.1016/j.btre.2021.e00592, 33537212 PMC7840853

[82] Bulus S. Antioxidant and biotechnological potential of *Pediococcus pentosaceus* RZ01 and *Lacticaseibacillus paracasei* RZ02 in a millet - based fermented substrate. Syst Microbiol Biomanuf. (2022) 3:571–84. doi: 10.1007/s43393-022-00126-3

[83] Given L. Phytochemical composition of *Lagenaria siceraria* fruits from KwaZulu-Natal and Limpopo, South Africa. Food Chem X. (2024) 22:101338. doi: 10.1016/j.fochx.2024.101338, 38623516 PMC11016956

[84] Hashemi SMB Jafarpour D Jouki M. Improving bioactive properties of peach juice using Lactobacillus strains fermentation: antagonistic and anti-adhesion effects, anti-inflammatory and antioxidant properties, and Maillard reaction inhibition. Food Chem. (2021) 365:130501. doi: 10.1016/j.foodchem.2021.130501, 34247050

[85] Hashemi SMB Jafarpour D. Fermentation of bergamot juice with *Lactobacillus plantarum* strains in pure and mixed fermentations: chemical composition, antioxidant activity and sensorial properties. LWT. (2020) 131:109803. doi: 10.1016/j.lwt.2020.109803

[86] Szutowska J. Functional properties of lactic acid bacteria in fermented fruit and vegetable juices: a systematic literature review. Eur Food Res Technol. (2020) 246:357–72. doi: 10.1007/s00217-019-03425-7

[87] Liu Y Wei Y Li H Li F Song M Li Z . Optimization of fermentation technology for composite fruit and vegetable wine by response surface methodology and analysis of its aroma components. RSC Adv. (2022) 12:35616–26. doi: 10.1039/d2ra04294k, 36545074 PMC9745641

[88] Meng J Wang J-L Hao Y-P Zhu M-X Wang J. Effects of *Lactobacillus fermentum* GD01 fermentation on the nutritional components and flavor substances of three kinds of bean milk. LWT. (2023) 184:115006. doi: 10.1016/j.lwt.2023.115006

[89] Prihanto AA Nurdiani R Jatmiko YD Firdaus M Kusuma TS. Physicochemical and sensory properties of terasi (an Indonesian fermented shrimp paste) produced using *Lactobacillus plantarum* and *Bacillus amyloliquefaciens*. Microbiol Res. (2021) 242:126619. doi: 10.1016/j.micres.2020.126619, 33189071

[90] Yang S Tao Y Maimaiti X Su W Liu X Zhou J . Investigation on the exopolysaccharide production from blueberry juice fermented with lactic acid bacteria: optimization, fermentation characteristics and Vis-NIR spectral model. Food Chem. (2024) 452:139589. doi: 10.1016/j.foodchem.2024.139589, 38744130

[91] Zhang Q Ma J Yang Y Deng J Zhu K Yi Y . Effects of S. Cerevisiae strains on the sensory characteristics and flavor profile of kiwi wine based on E-tongue, GC-IMS and 1 H-NMR. LWT. (2023) 185:115193. doi: 10.1016/j.lwt.2023.115193

[92] Kale P Mishra A Annapure US. Development of vegan meat flavour: a review on sources and techniques. Future Foods. (2022) 5:100149. doi: 10.1016/j.fufo.2022.100149

[93] Seo H Kim HR Cho IH. Aroma characteristics of raw and cooked *tenebrio molitor* larvae (mealworms). Food Sci Animal Resources. (2020) 40:649–58. doi: 10.5851/kosfa.2020.e35, 32734271 PMC7372989

[94] Li S Tang S He Q Gong J Hu J. Physicochemical, textural and volatile characteristics of fermented milk co-cultured with *Streptococcus thermophilus*, *Bifidobacterium animalis* or *Lactobacillus plantarum*. Int J Food Sci Technol. (2020) 55:461–74. doi: 10.1111/ijfs.14279

[95] Zhang R Song X Liu W Gao X. Mixed fermentation of *Chlorella pyrenoidosa* and *Bacillus velezensis* SW- 37 by optimization. LWT. (2023) 175:114448. doi: 10.1016/j.lwt.2023.114448

[96] Gomaa M Yousef N. Optimization of production and intrinsic viscosity of an exopolysaccharide from a high yielding Virgibacillus salarius BM02: study of its potential antioxidant, emulsifying properties and application in the mixotrophic cultivation of Spirulina platensis. Int. J. Biol. Macromol. (2020) 149:552–61. 32006575 10.1016/j.ijbiomac.2020.01.289

